# Influence of Surfactants with Differently Charged Headgroups on the Surface Propensity of Bromide

**DOI:** 10.1021/acs.jpca.4c07539

**Published:** 2025-03-21

**Authors:** Shuzhen Chen, Rawan Abouhaidar, Luca Artiglia, Huanyu Yang, Anthony Boucly, Lucia Iezzi, Jérôme Philippe Gabathuler, Thorsten Bartels-Rausch, Céline Toubin, Markus Ammann

**Affiliations:** † PSI Center for Energy and Environmental Sciences, Paul Scherrer Institut, 5232 Villigen, Switzerland; ‡ Department of Environmental System Science, ETH Zurich, 8093 Zürich, Switzerland; § Université de Lille, CNRS, UMR 8523—PhLAM—Physique des Lasers Atomes et Molécules, F-59000 Lille, France

## Abstract

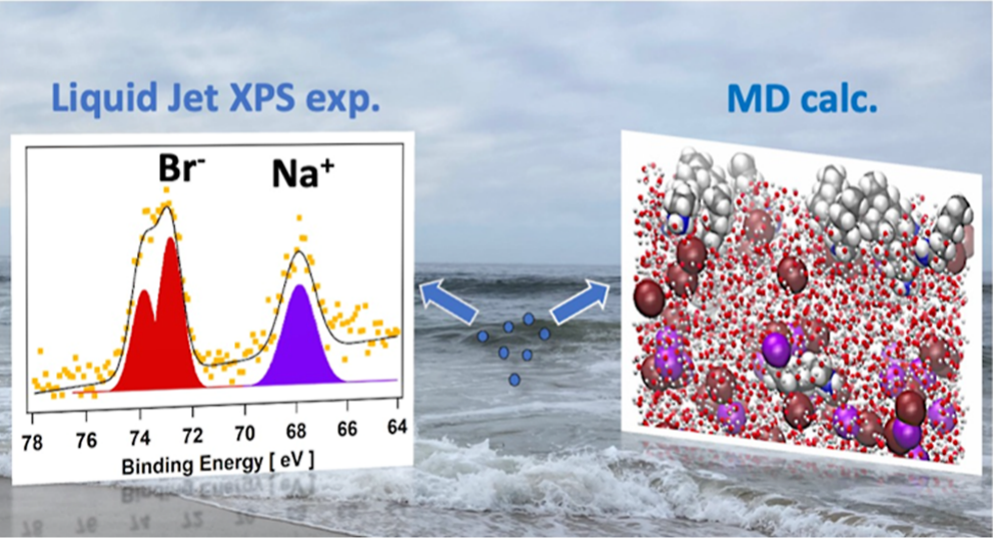

Halide ions in oceans and sea-spray aerosol particles are an important source of reactive halogen species in the atmosphere that impact the ozone budget and radiative balance. The multiphase cycling of halogen species is linked to the abundance of halide ions at the aqueous solution–air interface. Ubiquitously present surface-active organic compounds may affect the interfacial abundance of halide ions. Here, we use liquid jet X-ray photoelectron spectroscopy and molecular dynamics (MD) simulations to assess the impact of surfactants with different headgroups on the abundance of bromide and sodium ions at the interface. Core level spectra of Br 3d, Na 2s, and O 1s are reported for solutions containing tetrabutylammonium, hexylamine (HA), and propyl sulfate. We used a photoelectron attenuation model to retrieve the interfacial concentration of bromide in the presence of these different surfactants. The experimental results confirm the previously reported strong enhancement of bromide in the presence of tetrabutylammonium at the interface. In turn, propyl sulfate had a minor impact on the abundance of bromide but led to a significantly enhanced concentration of sodium cations. The MD simulations performed for bromide solutions containing hexylammonium and propyl sulfate show an enhancement of the interfacial bromide and sodium concentrations, respectively, comparable to the experimental results. The difference between the measured enhancement of bromide for HA and the nearly nonexistent effect of HA on bromide in the MD simulations is ascribed to the small amounts of hexylammonium present in the experimental solution. The present work suggests an important role of electrostatic interactions at the interface, which may guide the assessment of anion and cation abundances in atmospheric particles more generally.

## Introduction

Bromide ions in oceans and sea-spray aerosol (SSA) are an important source of bromine to the atmosphere, apart from bromine-containing halocarbons.^1^ On average, in seawater, the bromide concentration is about 8 × 10^–4^ M, a factor of 1.5 × 10^–3^ lower than that of chloride.^2^ Oxidation of bromide leads to reactive bromine species that, together with iodine and chlorine, initiate multiphase chemical and photochemical cycles that affect the ozone budget.^3^ The multiphase cycling of bromine species is affected by the abundance of bromide and other bromine species at the aqueous solution–air interface.^4−8^ While bromide in neat NaBr solutions is not enhanced at the interface,^6^ the reaction intermediate with ozone, a bromide ozonide, is surface-active.^5^ Also, the follow-up product, hypobromous acid, HOBr, shows some surface propensity,^6^ which is an essential intermediate in the formation of the molecular halogen compounds Br_2_ and BrCl. In mixed solutions, e.g., in the presence of NaCl, bromide experiences higher abundance at the surface than in pure NaBr solutions.^4^ In the presence of organic cosolutes, such as citric acid,^9^ butanol and butyric acid,^10^ and tetrabutylammonium,^11−13^ both attraction and repulsion of bromide have been observed. Some may be explained by ion pair formation or longer-range electrostatic interactions; but also, more subtle effects may govern the presence of bromide at the interface. The role of ion-pairing with surfactants has been suggested to determine the selectivity of ion–surfactant combinations in SSA from bubble bursting.^14,15^ Sodium–carboxylate ion pairs stabilize monolayers and increase their surface propensity.^16^ Especially for divalent ions such as Mg^2+^ and Ca^2+^, the interplay between hydration and ion-pairing with carboxylate ions is decisive for the selectivity suggested for SSA.^17,18^

Here, we follow up from previous work and extend the range of surfactants to obtain a more comprehensive understanding of the abundance of bromide ions at the aqueous solution–air interface at the ocean surface or in SSA particles. Neutral and ionic organic surfactants ubiquitously occur in the presence of marine biota and may get strongly enriched in SSA particles.^14,15,19−21^ In view of 71% of the earth’s surface being covered by oceans and in view of the large contribution of SSA to the global aerosol burden, an assessment of the impact of often surface-active organics on the interfacial abundance of bromide ions is important for understanding multiphase cycling of bromine. Alkylamines, such as tetrabutylamine and hexylamine (HA) used as proxies in this work, also originate more widely from other natural and anthropogenic sources.^19^ Similarly, organosulfate compounds, represented by propyl sulfate as a proxy in this work, are significantly contributing to atmospheric particulate organic matter, resulting mostly from multiphase processing of secondary organic compounds in the presence of SO_2_.^22−24^ Organosulfates, both through their hygroscopicity and surface activity, are also relevant as cloud condensation nuclei.^25^ The interplay of organosulfates with inorganic ions at the interface is therefore also relevant in that context.

X-ray photoelectron spectroscopy (XPS) provides chemically selective molecular level information from solid and liquid surfaces.^26^ With a tunable synchrotron X-ray source, the probe depth of the method can be adjusted from around one nanometer to a few nanometers by varying the kinetic energy (KE) of photoelectrons from a given core level via changing the exciting photon energy. Our recent work using liquid jet XPS demonstrated the power of this technique to assess the composition in terms of halide ions, organic solutes, and their mixtures at the aqueous solution–air interface.^9−11,27,28^ The photoemission signal provides a direct, chemically selective, spectroscopic signature from the surface and carries further information about acid dissociation, molecular orientation, and electronic structure.^26^

In our previous work, we have used liquid jet XPS^29^ to assess the impact of tetrabutylammonium on the interfacial abundance and reactivity of bromide, which was attributed to the positively charged headgroup ion-pairing with bromide, thus enhancing its interfacial abundance and reactivity with ozone. These experiments were performed at one single probe depth, thus leaving uncertainties with respect to the true bromide abundance at the surface. Here, we first extended that data set with KE (and thus probe depth)-dependent experiments. Next, we report experiments with neutral HA at high pH to attempt to examine the influence of the neutral amine on the abundance of bromide. To contrast these two, we also report XPS experiments with negatively charged propyl sulfate. Finally, we use classical molecular dynamics (MD) simulations to separately assess the role of HA, hexylammonium, and propyl sulfate on bromide and sodium cations at the interface and compare to experimental data.

## Experimental Methods

XPS experiments were performed using the near ambient pressure photoemission endstation consisting of a ScientaOmicron R4000 electron analyzer with a HiPP-2 pre lens and configured with a liquid jet chamber^29^ at the surfaces/interfaces: microscopy (SIM) beamline of the Swiss Light Source. For the present experiments, quartz nozzles (MicroLiquids) with diameters between 23 and 25 μm were used to deliver the liquid samples into the vacuum chamber. The liquid jet was operated at a flow rate of 0.5 mL/min, and the capillary transporting the liquid was embedded in a jacket until the feedthrough into the chamber. The jacket was connected to a cooling bath set to 277 K. Offline measurement indicated that the temperature of the liquid in the nozzle assembly was around 283 K, while the nozzle assembly (connector, translation stage) was connected to the chamber base at ambient temperature. After injection, the solution travels vertically downward with a laminar flow for a few hundred microseconds, corresponding to a few millimeters in length, before being hit by the X-ray beam. This time is sufficient for surface-active solutes at concentrations above 10^–2^ M to establish bulk–surface equilibrium,^30^ which has been proven by consistency between surface tension measurements and XPS derived surface composition.^28^ The temperature of the jet at that point may be a few K below the liquid temperature before the nozzle due to evaporative cooling once exposed to vacuum,^31^ though we assume the temperature is at around 283 K for the assessment of solution pH further below. The linearly polarized horizontal photon beam was 90° from the horizontal electron detection axis. The polarization vector was at an angle of zero° to the outgoing electrons. Correspondingly, we took into account the symmetry of the orbitals by applying the factor (1 + β(*h*ν))/4π to the ionization cross-section, where β(*h*ν) is the asymmetry parameter,^32^ remaining aware of the uncertainties of β in liquids.^33^ The electron analyzer was operated at 20 eV pass energy (for photoelectron KE at 155 eV) and 50 eV pass energy (for KE between 370 and 770 eV) with a sampling resolution of 0.1 eV energy steps. The KE was varied from 155 to 770 eV for obtaining core level spectra of Br 3d, N 1s, C 1s, Cl 2p, S 2p, and O 1s to allow variation of the information depth. Each KE corresponds to a specific value of the inelastic mean free path (IMFP) in liquid water.^34^ To take into account the 90° detection angle and the cylindrical liquid filament geometry, the mean escape depth (MED) of photoelectrons was obtained as MED = (2/π) IMFP.^30^

Reported binding energies are relative to the vacuum level and calibrated to the O 1b1 orbital of liquid water at 11.3 eV,^35^ measured for each solution and each photon energy, as well as for a 0.05 M NaCl solution used as purging solution when switching from one solution to the next. This was also used to check the grounding after each cleaning of the chamber and nozzle assembly. The spectral region was fit using pure Gaussian functions following standard linear background subtraction. The error bars of reported spectra were evaluated based on the standard deviation calculated from the fits of a statistical sample of spectra corresponding to the same solution. The standard deviation values were propagated to obtain the error of the signal intensity ratios. This does not take into account either the small movements of the liquid filament in front of the aperture of the analyzer or the uncertainty in the measurement of the photon flux.

## Materials

This study was conducted using sodium bromide (NaBr, Sigma-Aldrich, >99.0%), sodium chloride (NaCl, Sigma-Aldrich, >99.0%), tetrabutylammonium (TBAH^+^) bromide (TBA-Br, Sigma-Aldrich, >99.0%), HA (CH_3_(CH_2_)_4_CH_2_NH_2_, Sigma-Aldrich, >99.0%), and sodium propyl sulfate (PS^–^) (CH_3_CH_2_CH_3_OSO_3_Na, CAS no. 1000-56-2, Chembo Pharma Ltd., >98.0%). The solutions were prepared by adding stock solutions to Milli-Q water (Millipore, 18.2 MΩ cm at 25 °C) and degassing with Ar gas. 0.1 M NaBr, 0.1 M TBA-Br, 0.1 M TBA-Br/0.55 M NaCl, 0.1 M NaBr/0.1 M HA, 0.1 M NaBr/0.1 M HA/0.55 M NaCl, 0.1 M NaBr/0.1 M sodium PS^–^, and 0.1 M NaBr/0.1 M sodium PS^–^/0.55 M NaCl aqueous solutions were prepared for the liquid jet XPS experiment. The pH of the 0.1 M NaBr/0.1 M HA and 0.1 M NaBr/0.1 M HA/0.55 M NaCl solutions at *T* = 21 °C was 12.2 and 12.5, respectively. The pH values of the reference solutions of HA of 12.2, 10.2, and 8.2 were adjusted by adding NaOH and H_2_SO_4_ with the help of an Orion pH meter with the Orion Ross 8103BN semimicro pH electrode.

## Theoretical Calculations

For better insights into the surface propensity of the ions at the air/liquid interface, MD simulations of 1.00 M NaBr, 0.50 M NaBr/0.50 M hexylammonium (HAH^+^) bromide, 1.00 M NaBr/0.50 M HA, and 1.00 M NaBr/0.50 M sodium propyl sulfate with and without 1.00 M NaCl were performed using the GROMACS program suite.^36^ For HAH^+^ and HA, molecular interaction parameters were based on the all-atom optimized potentials for liquid simulations (OPLS/AA) force field.^37^ Water was described with the SPC/E model,^38^ which is widely utilized in bulk simulations.^39^ For the PS^–^ ion, we used force field parameters benchmarked against molecular simulations of common ionic liquids.^40^ This combination of the OPLS/AA force field and SPC/E water has been validated in earlier studies.^10,39^ Additionally, partial charges for the simulations were derived using the restrained electrostatic potential approach,^41^ which relies on quantum calculations at the B3LYP/6-311+G(d) level using the Gaussian “16.C.01” software (Supporting Information Section S3, Table S2). Finally, nonpolarizable force field parameters developed by Horinek et al.^42,43^ were used for the inorganic ions (Na^+^, Br^–^, and Cl^–^). This set of parameters has shown good agreement with experimental data and polarizable models.^44,45^

The number of ion pairs used in each simulation depends on the mixture, the mixing ratios of the simulated systems being chosen to be in the range of the experimental conditions (Table S1) and to ensure charge neutrality of the solution. The ions and the water molecules were placed in a cubic box of dimension 70 Å. Periodic boundary conditions were applied in all three dimensions. We used a 1.2 nm cutoff for short-range interactions combined with the particle mesh Ewald summation method with a relative tolerance of 10^–5^, fourth-order cubic interpolation, and a Fourier spacing parameter of 0.15 to account for the long-range Coulomb interactions.^46^ Bonds are constrained using the LINCS algorithm,^36^ enabling the use of a 2 fs time step. The temperature is held constant at 280 K using a Nosé–Hoover thermostat,^47^ with a coupling constant of 0.4 ps. This temperature was chosen to reproduce the experimental conditions, 10 K below ambient temperature.

All initial system geometries were first optimized through the steepest descent algorithm. This was followed by an equilibration in the isothermal–isobaric (*NPT*) ensemble at 1 atm with a Berendsen pressure coupling for 500 ps. The equilibrated bulk sample is then placed into the center of a parallelepiped box that was elongated along the interfacial normal direction (*z* axis), with final dimensions of 70 Å × 70 Å × 140 Å. This allows for the presence of two liquid–vacuum interfaces above and below the liquid slab. Then, a 5 ns annealing in the isothermal–isochoric (*NVT*) ensemble was performed, warming the system to 400 K and then cooling down to 280 K to enable all components to diffuse into the system. Finally, the *NVT* ensemble trajectory was executed at 280 K for 150 ns for each mixture. The molecular structures and configurations along the MD simulations are visualized via VMD software.^48^

To quantify molecular properties at interfaces, it is essential to compute averages based on the positions and orientations of molecules relative to the interface. Distinguishing between mean and instantaneous interfaces^49^ may be relevant in such analyses, as also suggested for the quantitative interpretation of photoemission signals.^50^ However, in the present study, comparisons are made between different mixtures, with surface propensity defined within the framework of an average interfacial region across the Gibbs dividing surface, and the focus is on understanding the average effect of the surfactants on the surface propensity of bromide rather than dynamic effects. Limitations of our approach are addressed in the Discussion section.

## Results

**Figure 1:**
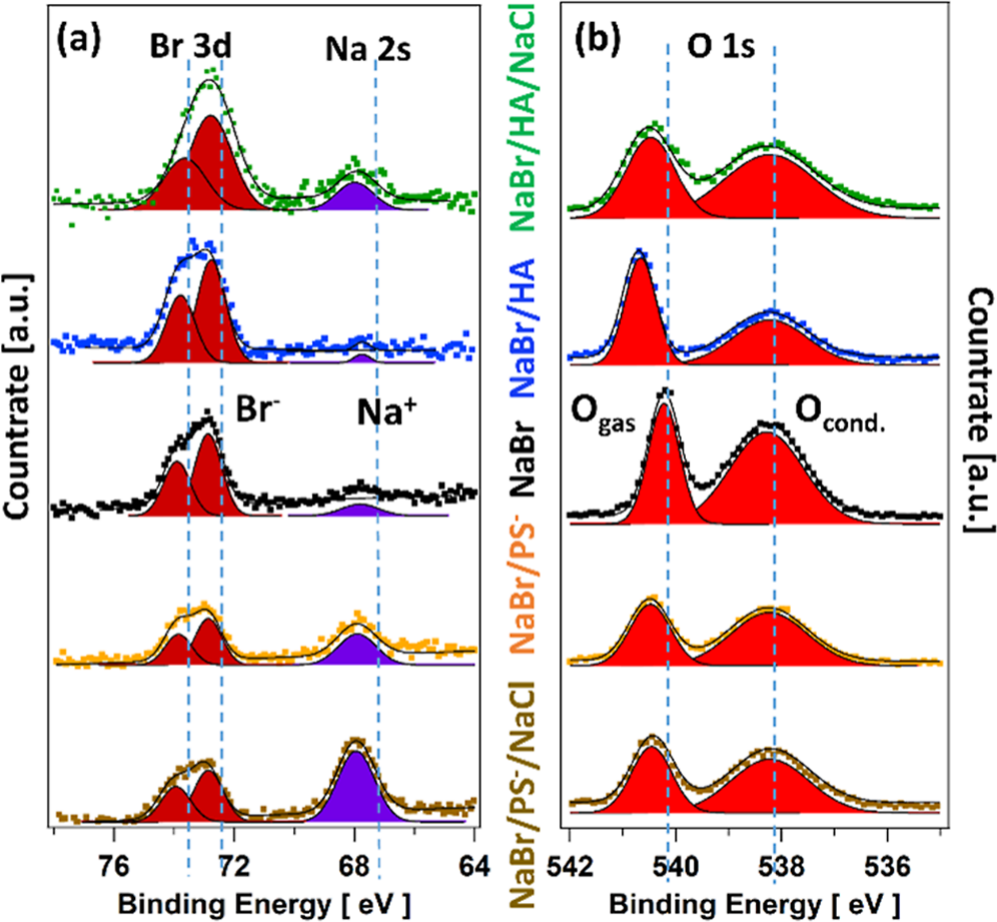
(a) Br 3d and Na 2s and (b) O 1s spectra (not normalized, same *y*-scale) of mixed 0.1 M NaBr/0.1 M HA/0.55 M NaCl (pH 12), mixed 0.1 M NaBr/0.1 M HA (pH 12), 0.1 M NaBr, mixed 0.1 M NaBr/0.1 M sodium propyl sulfate (PS^–^) (neutral pH), and mixed 0.1 M NaBr/0.1 M sodium PS^–^/0.55 M NaCl (neutral pH) aqueous solutions at a photoelectron KE of 155 eV.

Figure 1 shows the measured Br 3d, Na 2s, and O 1s photoemission count rates for the NaBr solution and its mixture with HA and propyl sulfate (PS-) without and with additional NaCl, respectively. Corresponding spectra of tetrabutyl ammonium bromide (TBA-Br) were already reported in our previous work by Chen et al.^11^ The spectra were measured at photon energies of 229 eV for Br 3d and Na 2s and 696 eV for O 1s to obtain the same KE of around 155 eV, corresponding to a MED of 0.6 nm. Panel a shows Br 3d (dark red) and Na 2s (purple) spectra, while panel b features the O 1s (red) photoelectron spectra. In panel a, the higher binding energy portion of the spectra shows the spin–orbit split Br 3d_5/2_ and 3d_3/2_ peaks (dark red), while the lower binding energy portion features the Na 2s peak (purple) at 67.9 eV of Na^+^ ions in the solutions. In panel b, the O 1s spectra reflect the oxygen of gas-phase H_2_O at higher binding energy and of liquid H_2_O at lower binding energy. The binding energy differences between O 1s liquid and gas are different for the different solutions, likely due to differences in surface potentials, as observed in previous studies.^10,11,51^ The organic free reference of 0.1 M NaBr (neutral pH, black) is shown in the center, its mixture with 0.1 M HA (pH 12.2, blue) as the first row above the center, and with additional 0.55 M NaCl (pH 12.5, green) in the top row on both panels. In turn, the mixture of 0.1 M NaBr with 0.1 M sodium PS^–^ (neutral pH, yellow) is shown in the first row below the center and with additional 0.55 M NaCl (neutral pH, brown) in the bottom row.

For 0.1 M TBA-Br, thus, in the presence of the positively charged tetrabutyl ammonium (TBAH^+^), Chen et al.^11^ observed a marked increase of the Br 3d intensity in comparison to that of NaBr. Here, upon addition of the supposedly neutral HA, shown in the top two spectra of Figure 1a, the Br 3d peak area increases somewhat compared to that of pure NaBr solutions. In contrast, upon addition of sodium PS^–^, the Br 3d peak area decreases (bottom of Figure 1a). Compared with pure NaBr, in the presence of 0.1 M HA, the sodium intensity is barely detectable (top of Figure 1a). Note that the increased Na 2s intensity for solutions in the presence of 0.55 M NaCl is due to the 5-fold increase of the bulk concentration of sodium. Upon adding sodium PS^–^, we observe a strong increase of the Na 2s intensity by more than expected from the factor of 2 increase of the bulk sodium cation concentration. The further increase of the Na 2s signal in the presence of additional 0.55 M NaCl then reflects the additionally added sodium cations.

Note that the changes in photoemission intensity among the various solutions are not only due to the different abundances of Br^–^ and Na^+^ at the interface but also affected by the attenuation of photoelectrons by the aliphatic carbon chains accumulating at the interface, especially at the low MED at 155 eV KE of 0.6 nm. This is also apparent in the condensed phase O 1s peak, which is the highest for NaBr (black, center of Figure 1b) and lower in the presence of the added organics. Thus, for Br 3d, the increase in intensity in the presence of HA is partly offset due to the attenuation by HA on the surface. Consistent behavior was also observed with TBAH^+^.^11^

Both, the O 1s and Br 3d spectra in Figure 1 show a significant broadening of peaks in the presence of NaCl. We observed (but not explicitly described or discussed) similar effects also with the TBAH^+^ solutions^11^ and with other surfactants.^52^ Since this is also apparent in the gas-phase O 1s peak, this seems to be related to variability or fluctuations of the surface potential, possibly induced by an increase of the amount of organics at the interface driven by salting out effects in the presence of 0.5 M NaCl. Note that as described in the Experimental Methods section, the grounding was routinely checked by measuring the O 1b1 orbital of 0.05 M NaCl purge solutions between the solutions reported here.

C 1s, N 1s, S 2p, and Cl 2p spectra are shown and described in the Supporting Information (Section S1, Figure S1). The N 1s spectrum is dominated by the neutral amine, exhibiting a 5% share of HA, while we would expect an about 11% contribution of hexylammonium in the bulk, though with some uncertainty due to the uncertainty in the liquid jet temperature and the related response of the pH (see Supporting Information Section S1). Still, the measured spectrum indicates that the hexylammonium is present to a smaller degree at the interface than expected based on the bulk equilibrium and similar to previous studies.^53^

In order to allow quantification of the effect of the different surfactants TBAH^+^, HA, and PS^–^ on the abundance of bromide at the interface, we measured the Br 3d and O 1s spectra at different KEs to obtain photoemission intensities with different probe depths. For TBAH^+^, in our previous work,^11^ we have quantified the surface coverage for the solution concentrations also used here to be at around 3 × 10^14^ cm^–2^. Here, we have not included further measurement of C 1s spectra in this series, neither as a function of the surfactant concentration nor as a function of KE that would be required for reliable surface coverage retrieval and comparison to surface tension measurements.^10,11^

**Figure 2:**
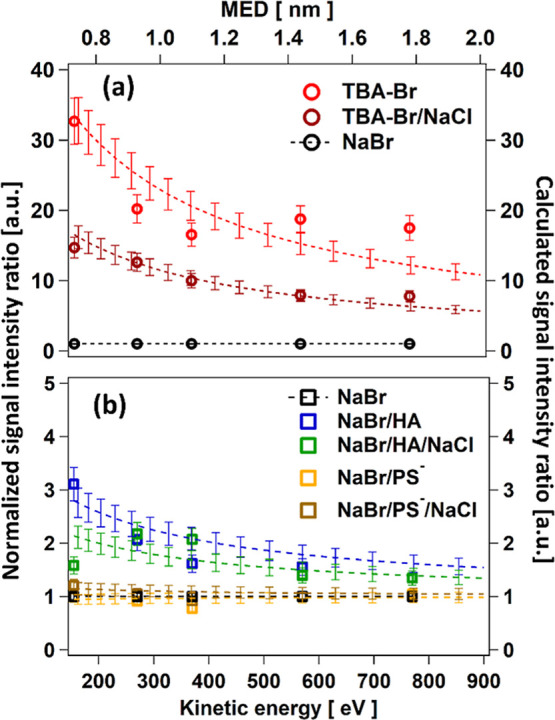
Signal intensity ratios of Br/O for (a) 0.1 M TBA-Br and mixed 0.1 M TBA-Br/0.55 M NaCl and for (b) mixed 0.1 M NaBr/0.1 M HA, mixed 0.1 M NaBr/0.1 M HA/0.55 M NaCl, mixed 0.1 M NaBr/0.1 M sodium PS^–^, and mixed 0.1 M NaBr/0.1 M sodium PS^–^/0.55 M NaCl aqueous solutions as a function of electron KE, after normalization to the corresponding intensity ratio for the 0.1 M NaBr solution, photon flux, and cross-section. Dashed lines are fits of the attenuation model to the data. Their error bars reflect the uncertainties related to the parameters characterizing the neat NaBr solution (see the text in the Discussion section).

Figure 2 shows the normalized photoemission signal intensity ratio of Br 3d to condensed phase O 1s (a) as a function of the KE to obtain its variation with the photoelectron MED. Normalization was done by the cross-section and the photon flux. Taking the ratio of the normalized signals of two elements measured at the same KE removes the sensitivity of the signals to KE-dependent factors of the analyzer and to the geometric sample configuration. Further normalization of these ratios to those of neat NaBr removes uncertainties in the photon flux. Assuming that the aliphatic carbon layer formed by the surfactants attenuates photoelectrons from Br 3d and O 1s in the same way, the first normalization also removes the effect of this attenuation. The doubly normalized ratios indicate how the amount of interfacial bromide on the aqueous side of the interface changes in response to a surfactant being present. Figure 2a shows that the Br/O signal intensity ratio is strongly enhanced in the presence of TBAH^+^ in comparison to that of the neat NaBr solution, also when NaCl is present in addition. The enhancement decreases with MED, thus, when a higher fraction of the signals are derived from deeper in the bulk. Figure 2b shows the corresponding results for HA and PS^–^. Obviously, the Br/O ratio is still significantly enhanced in the presence of HA, though about an order of magnitude less than with TBAH^+^. And also, in this case, the relatively lower enhancement in the presence of 0.5 M NaCl is comparable, even though we note that the scatter is higher in the presence of NaCl due to the broadening of the spectra mentioned and discussed above, which might have especially affected the data point at the lowest KE. The Br/O ratio is not significantly affected by the presence of PS^–^. The values of the measured Br/O ratios at the lowest KE are also reported in the first column of Table 1. The dashed lines in Figure 2 are the fit results using an attenuation model introduced further below. The scatter in the data for Na 2s signals as a function of KE was substantial. While the measurements at the other KEs were qualitatively in line with the enhanced Na 2s signals in the presence of sodium PS^–^ observed at the lowest KE (Figure 1), we did not further analyze them to retrieve interfacial concentrations.

**Table 1:** Comparison of Normalized Photoemission Signal Intensity Ratios and Surface to Bulk Concentration Ratios Derived from XPS and MD Simulations^e^^,^^g^

solutes	pH^a^	[*I*_Br 3d_/*I*_O 1s_]_norm_^b^^,^^d^ (XPS)	[*n*_Br,Δ_/*n*_Br,b_]_norm_^c^^,^^d^ (att. mod.)	[*n*_Br,Δ_/*n*_Br,b_]_norm_^c^^,^^d^ (MD)	[Org.]_i_/[Org.]_b_^f^
NaBr	7	1	1	1	
NaBr/NaCl	7			1.66	
NaBr/TBAH^+^	7	33	352		
NaBr/NaCl/TBAH^+^	7	15	168		
NaBr/HAH^+^				4.77	2.90
NaBr/NaCl/HAH^+^				4.47	4.99
NaBr/HA	12	3.1	20.0^f^	1.31	
NaBr/NaCl/HA	12	1.6	12.9^f^	1.52	
NaBr/PS^–^	7	0.99	0.0	0.91	1.57
NaBr/NaCl/PS^–^	7	1.2	2.2	1.20	3.23

a pH values indicated in this column are the values of the experimental solutions at 294 K.

b *I*_Br 3d_ and *I*_O 1s_ are the normalized photoemission signal intensities derived from the XPS spectra shown in Figure 1 (and taken from Chen et al.^11^ for TBAH^+^) and used in Figure 2.

c *n*_Br,Δ_: number of Br atoms per unit volume in the surface layer with thickness Δ = 0.5 nm; *n*_X,b_: number of Br atoms per unit volume in the bulk phase; both from the attenuation model.

d The ratios are normalized to those for the pure NaBr solution.

e The XPS experiments were performed at a pH around 12, and HAH^+^ was a 5% fraction (Supporting Information Section S1). HAH^+^ was absent in the MD simulations for HA.

f [org]_i_ and [org]_b_ refer to the interfacial and bulk concentration (in M) of the surfactants. Their ratio is given in this table. For HA, the number of bulk phase molecules after equilibration was insufficient to accurately determine a meaningful bulk concentration.

g TBAH^+^ = tetrabutylammonium; HA = hexylamine; HAH^+^ = hexylammonium; PS^–^ = propyl sulfate.

**Figure 3:**
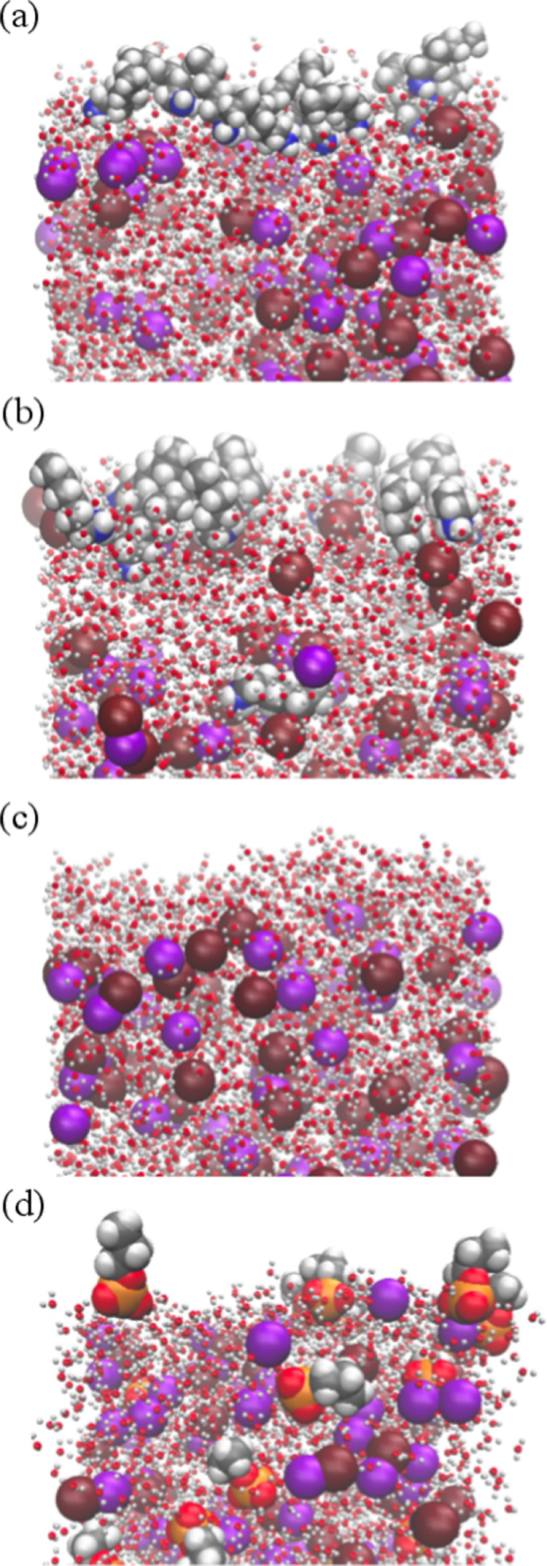
Snapshots illustrating the composition of mixed sodium bromide solutions under various conditions: (a) with HA, (b) with HAH^+^, (c) pure NaBr, and (d) with PS^–^. Na^+^ ions stand in violet, Br^–^ ions in dark red, and aliphatic carbon atoms in gray. He headgroups of HAH^+^ and HA are in dark blue, while that for PS^–^ is in yellow, respectively.

**Figure 4:**
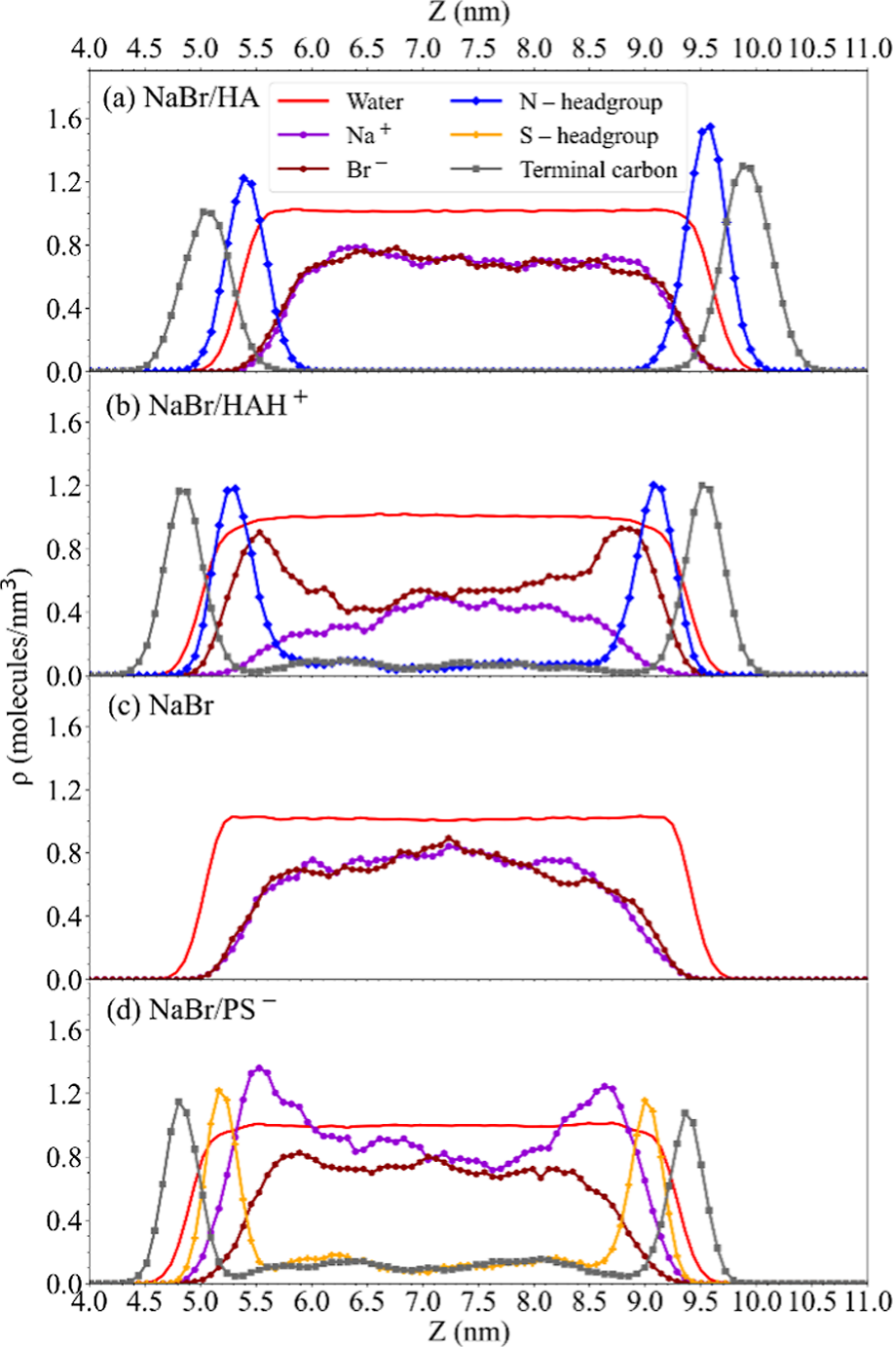
Atomic density profiles calculated from MD simulations of (a) mixed 1 M NaBr/0.5 M HA, (b) mixed 0.5 M NaBr/0.5 M hexylammonium (HAH^+^) bromide, (c) 1 M NaBr, and (d) mixed 1 M NaBr/0.5 M sodium propyl sulfate (PS^–^) aqueous solutions. Densities of water molecules are shown in red, Na^+^ ions in violet, Br^–^ ions in dark red, terminal carbon chains in gray, and the headgroups of HAH^+^and HA are in dark blue, and that of PS^–^ are in yellow, respectively. To seek clarity, water density has been scaled (divided by a factor 30) so that we can more easily identify the bulk and interface regions.

Figure 3 showcases MD snapshots of NaBr solution (c) and its mixture with HA (a), HAH^+^ (b), and PS^–^ (d). The composition of the interfacial layer can be characterized by the density profiles of the atoms of interest along the direction normal to the interface, i.e., the *z*-axis. Figures 4 and S4 depict the density profiles for the different components for the solutions without and with additional NaCl, respectively. These profiles are computed in terms of the number of molecules per unit volume (molecules/nm^3^) and are plotted against z (nm). The profiles are shown for water oxygen atoms, ions (Na^+^, Br^–^, Cl^–^), and the head groups (N and S) associated with both –NH_2_ and –NH_3_^+^ functionalities, as well as –OSO_3_^–^ ions and their respective terminal carbon atoms. For all solutions, the water density profile is reported. It is characterized by a constant profile in the bulk region, while the interfaces correspond to the region where the water density drops. Figures S5 and S6 show corresponding relative density profiles for each ion, again without and with additional NaCl, respectively. Starting with the reference system, the pure NaBr (Figure 4c, second row from bottom) solution, the simulations show an almost flat profile in the bulk region and depletion toward the interface with a marked drop of the bromide and sodium densities, discussed further below. For surfactants-containing solutions, the surfactants strongly partition to the interface, as depicted by the pronounced peaks in the densities on either sides of the water film. The bulk is also significantly depleted at equilibrium with small oscillations in the densities, or nearly no density in the case of HA. The surfactant head groups are located toward the water phase, with the charged head groups [ammonium (b) and sulfate (d)] being more integrated into the water surface layer. This is in contrast to the neutral amine (a), which is more dispersed at farther distances (z) from the two interfaces. The terminal carbons reside well above the interface, resulting in more distant peaks with respect to the water density. In the presence of HAH^+^ (Figure 4b), the bromide density shows maxima at the interface just underneath the ammonium group. Conversely, in the presence of PS^–^, sodium ions exhibit a similar behavior. With HA, the density of bromide at the interface is also somewhat larger than for the pure NaBr solution but far less than with HAH^+^. In the presence of NaCl (Figures S4 and S6), bromide and chloride share very similar profiles, and both together exhibit weaker relative accumulation at the interface than bromide alone. The local environment of each component is reported in terms of their radial distribution functions (RDFs) provided in Figures S7 and S12 and discussed further below.

In order to allow comparison with the XPS data further below, density profiles of ions or molecules were integrated within the range of 10% to 90% of the water bulk density, the commonly used interfacial region for aqueous solutions^54^ (detailed results in Table S3). This leads to a 0.5 nm wide interfacial region on both sides of the liquid slab (Table S4). Interfacial concentrations given in Table S3 are given in units of M (number of molecules or ions within the 0.5 nm thick layer divided by its volume) and in units of molecules cm^–2^ (number of molecules or ions in the same layer divided by its surface area). The bulk density of the same species was computed by integrating the region between these two interfaces. This integration was performed across multiple time intervals for the last 50 ns of the trajectory: 95 to 100 ns, 115 to 120 ns, 135 to 140 ns, and 145 to 150 ns. The results are also reported in Table S3. The final bulk concentrations depend on both the initial molecular composition of each mixture and the surface affinity, which causes bulk depletion. This is a major difference from the experiment. For the liquid jet experiments, for the conditions and concentrations applied in this work,^11,30^ accumulation or depletion of a species at the surface does not lead to a change in the bulk liquid phase of the liquid filament due to its 20 μm diameter in contrast to the 5 nm liquid slab of the simulations. Thus, the equilibrated surface and bulk concentrations of the simulations are not the same as those in the experiment. We therefore report the ratios of interfacial to bulk concentration, [Br]_i_/[Br]_b_ and [Na]_i_/[Na]_b_, also after normalization to the corresponding ratios for pure NaBr solution (blue shaded columns in Table S3), which leads to representation of the theoretical results corresponding to those reported for the XPS data in Figure 2, though limited to those for bromide.

The overall NaBr concentration (1.00 M) was higher than that in the experiments to obtain better statistics. After equilibration of the pure NaBr solution, the average bulk density of Na^+^ and Br^–^ was 1.09 M, thus higher than the average overall concentration due to the depletion at the interface. As apparent from Table S3, the interfacial Na^+^ and Br^–^ concentrations are 0.06 ± 0.01 M, thus just about 6% of that in the bulk. In turn, for the surfactants, the bulk–surface equilibrium drives them to the surface and due to the limited number of molecules in the simulation box, as just mentioned above, the bulk concentration gets very low, particularly in the case of HA. The bulk concentration of HA evolved from 0.5 M at the start to 0.05 M after equilibration, that of HAH^+^ from 0.5 to 0.31 M, and that of PS from 0.5 M to 0.39 ± 0.01 M. For instance, on average, only 2 molecules of HA remained in the bulk, but 14 molecules remained in the case of HAH^+^. The interfacial concentrations are 5.68 × 10^13^ molecule cm^–2^ (1.89 ± 0.02 M), 2.74 × 10^13^ moleculecm^–2^ (0.91 ± 0.02 M), and 1.82 × 10^13^ (0.61 ± 0.05 M) molecules cm^–2^, respectively, which are generally an order of magnitude below the maximum (monolayer) coverage commonly observed for organic surfactants of around 3 × 10^14^ molecule cm^–2^ on an aqueous droplet.^55^ Interfacial to bulk concentration ratios are also reported in Table 1, except for HA due to the low number of HA molecules remaining in the bulk. As mentioned above, we have not attempted to quantify the surface coverage of the surfactants from the XPS data to compare with.

For facilitating the comparison of the integrated density profiles for bromide derived from the MD simulations with the XPS results, we applied a simple attenuation model developed previously^10,11^ and described and discussed in detail in the Supporting Information Section S2. It is used to calculate photoemission signals as a function of KE, as shown in Figure 2. It is based on an assumed density profile and takes into account attenuation of photoelectrons originating from different depths. Similar to the presentation of the XPS data above, we assume that the surfactant layer on the top of the aqueous phase is affecting the Br 3d and O 1s signals in the same way. In essence, the model assumes that a layer of thickness Δ at the top of the aqueous phase exhibits a bromide concentration, *n*_Br,Δ_, enhanced or reduced from that in the bulk, *n*_Br,b_, by a factor *f* such that *n*_Br,Δ_ = *f n*_Br,b_. We choose the same interfacial thickness as for the integration of the density profiles obtained from the MD simulations: Δ = 0.5 nm. The calculated Br/O signal intensity ratios were fitted to the measured ratios by varying *f*, shown as dashed lines in Figure 2. The detailed parameters are reported in Table S1, which can be directly compared to the corresponding values obtained from the MD simulations and are reported in Table S3. As a summary, the normalized interface to bulk concentration ratios are provided in Table 1. These ratios are about an order of magnitude higher than those obtained by XPS. This is consistent with the fact that for the attenuation model, the interfacial thickness was assumed to be 0.5 nm, while the photoemission signals integrate over about one nm at the lowest KE.

The TBAH^+^ bromide solutions, with all headgroups fully protonated at neutral pH, attract much more Br^–^ into the interface, leading to a 35-fold enhanced interfacial bromide concentration and a 17-fold enhancement in the presence of 0.5 M NaCl and, thus, when normalized to the 0.1 M NaBr solution, to the factors of 352 and 168, respectively. The experimental enhancements of bromide in the presence of TBAH^+^ are best compared to the ones provided by the MD simulations for the fully protonated HAH^+^, which were 4.8 and 4.5, respectively. When comparing, we remain aware that the HAH^+^ surface coverage in the simulation was around an order of magnitude lower than that of TBAH^+^ in the experiment.

In the presence of HA, the interfacial concentration of Br^–^ derived from the XPS data is about a factor of 2 higher than in the bulk, which is a factor of 20 higher than in the case of the pure NaBr solution (Table 1). This is a bit more than an order of magnitude less than that for TBAH^+^. When 0.55 M NaCl is present together with 0.1 M NaBr and 0.1 M HA, the interfacial concentration of Br^–^ is about 30% higher than that in the bulk, which is a factor of 13 higher than the pure NaBr Br intensity. In the presence of HA, only a slight interfacial enhancement is derived from the simulations with 1.3 for pure NaBr and 1.5 for NaBr/NaCl solutions, thus in some contrast with the experimentally measured enhancement for HA. In the presence of sodium PS^–^, the fits to the XPS data indicate a strong interfacial depletion by more than an order of magnitude in comparison to that of pure NaBr, while the value from the MD simulations (0.91) indicates a smaller effect (Table 1). In turn, in the presence of additional 0.55 M NaCl, the interfacial bromide concentration derived from XPS exhibits an opposing trend, a factor of 2.2 higher than for the pure NaBr solution. At least consistent with the increasing trend, MD results indicate a 1.2-fold increase. We caution that the Br 3d intensities in the presence of PS^–^ were low overall and the NaCl-containing solution showed significant broadening of the XPS spectra that made fitting and quantifying more difficult.

For sodium, from the MD simulations, suppression was observed with HAH^+^ (0.7 and 0.8, respectively) but enhancement in the presence of HA (1.4 and 1.3, respectively). In the presence of PS^–^, based on the measurement presented in Figure 1, the Na 2s signal intensity was higher than that in the presence of HA, also in the presence of NaCl. But, as mentioned above, the KE-dependent data showed too much scatter to allow reliable interfacial concentration retrieval as for bromide. From the MD simulations, the ratio to the pure NaBr solution is 2.6. Notably, the presence of NaCl has no impact on the abundance of sodium.

## Discussion

For pure NaBr solutions, MD simulations indicate that neither Na^+^ nor Br^–^ exhibits a preferential surface affinity (Figure 4c). In these solutions, the average bulk density is 1.09 M, while the interfacial Na^+^ and Br^–^ concentrations amount to only 6% of their bulk values. This level of depletion aligns with previous studies utilizing nonpolarizable models.^6,42,43,56,57^ As discussed in the context of calculating photoemission signals in the Supporting Information Section S2, this depletion is consistent with reported surface tension. Since we focus on the impact of the surfactant headgroup charge in this work and since we present the experimental data normalized to the pure NaBr solution case, we refrain from reiterating the recent debates around the abundance of halide ions at the interface of pure aqueous halide solutions based on theory and experiment.^4−6,43,58−69^ In general, the abundance of halides decreases with decreasing halide ion size, in agreement with MD simulations,^70^ surface tension results,^70,71^ and XPS.^72^

Still in the absence of organics, the presence of 1.0 M NaCl affects the abundance of bromide ions at the interface. Specifically, the interfacial concentration of bromide increases from 0.06 ± 0.01 M to 0.11 ± 0.02 M, indicating a salting-out effect of bromide in the presence of chloride. The bromide-to-chloride concentration ratio is approximately 50% higher at the surface, likely due to the larger size and higher polarizability of Br^–^, which makes the interaction with the surface region more favorable for bromide and ion depletion at the surface more pronounced for chloride than for bromide. We have not measured spectra from mixed NaBr/NaCl solutions to compare with these as part of this work. Previous XPS experiments have shown some enhancement of the interfacial bromide to chloride ratio.^4^Figure S9 illustrates the RDFs of anion– and cation–water interactions. A peak at 0.25 nm for the Na^+^–O_W_ pair suggests a close association between sodium ions and water molecules. For the Br^–^–O_W_ and Cl^–^–O_W_ pairs, the hydration shell radii are approximately 0.33 and 0.32 nm, respectively. These findings align with previous work.^73,74^

Considering the interfacial to bulk concentration ratios derived from the MD simulations for the two ionic surfactant species for the NaBr solutions (Tables S3 and 1), as expected, the HAH^+^ ([org]_i_/[org]_b_ = 2.90) with the longer alkyl chain is more surface-active than PS^–^ ([org]_i_/[org]_b_ = 1.57) driven by the number of hydrophobic interaction options. And when considering the charge state at an identical alkyl backbone, the neutral HA is much more surface-active than the charged HAH^+^, which complicates the estimation of its bulk concentration. While we have not attempted at quantify the surface coverages from the XPS data, the C 1s spectra (Figure S1) qualitatively confirm this picture. The intensity for the HA solution is much higher than that for the PS^–^ solution. The ratio is roughly an order of magnitude higher than expected based on the carbon number difference (note the scaling factor for the C 1s spectrum of propyl sulfate). As mentioned in the Results section and discussed in more detail in Supporting Information Section S1, the N 1s spectrum indicates that the fraction of hexylammonium at the surface is smaller than expected for the bulk, also when taking into account the uncertainty of the liquid jet temperature. This supports the higher surface propensity of HA than that of HAH^+^. The generally larger surface propensity of neutral acids or bases than their conjugate ionic bases or acids has been well documented by theory and experiment.^28,53,75−78^ The surface activity of the organic solutes is strongly driven both by the hydration of their hydrophilic headgroups and by the increasing hydrophobic nature of their alkyl chains as these chains lengthen, as observed for monocarboxylate and dicarboxylate ions.^75,76^Figure S8 shows the RDFs for the three organic solutes with water. The strong Coulombic interaction develops between the HAH^+^ cation and water through the formation of N–H–O_W_ hydrogen bond-like bonding at 0.18 nm. For HA molecules, the peak is less pronounced, and it is slightly displaced to 0.2 nm. For PS^–^, sulfate ions interact also strongly with water molecules through the hydrogen atoms, forming a S–O–H bond, which is why the pair distribution function peaks at a larger distance (around 0.28 nm) than for HAH^+^.

Table 1 also shows the surface-to-bulk ratio of each organic, with and without 1 M NaCl, derived from the MD simulations. In the presence of NaCl, the surface-to-bulk molar ratio is higher than without salt for HAH^+^ ([org]i/[org]b = 4.99 vs 2.90) and PS^–^ ([org]i/[org]b = 3.23 vs 1.57). This indicates salting effects of approximately 1.7 and 2.0 for HAH^+^ and PS^–^, respectively. In the case of HA, the low bulk concentration results in a correspondingly high ratio, where almost no salting out effect is apparent. From the XPS data, the salting effect is best seen with the N 1s spectrum for HA and the S 2p spectrum for PS^–^ (Figure S1), which both increase in intensity in the presence of NaCl, though this is somewhat confounded by the broadening of the spectra mentioned already above. A similar effect was also observed in the TBAH^+^ spectra already shown in our previous work.^11^ The increasing surface coverage of organic solutes with increasing salt concentration driven by increasing partitioning to the air–liquid interface due to the salting out effect is known for many species.^79^

The main focus of this study has been to compare the surfactant-containing solutions with the neat NaBr solutions experimentally with XPS in terms of spectra in Figure 1 and signal intensity ratios as a function of MED in Figure 2, along with the concentration ratios in Table 1 obtained from the attenuation model, and in terms of the density profiles from the MD simulations, Figure 4, along with the integrated concentration ratios in Table 1. From the experiments, the directly apparent results are that Br^–^ is strongly pulled toward the surface in the presence of TBAH^+^ and to a much smaller degree in the presence of HA. Na^+^ is doing so in the presence of PS^–^. Based on the MD simulations, the enhancement of bromide in the presence of a positively charged surfactant is demonstrated for HAH^+^ and that of sodium for PS^–^. In the simulations, the surface concentrations of both ions are only slightly enhanced (factors 1.31 and 1.37 for Br^–^ and Na^+^, respectively) in the presence of the neutral HA. Even though the measured fraction of HAH^+^ at the surface of the experimental HA solutions was only 5%, it may have been sufficient to exert the observed level of enhancement of bromide. While this enhancement was more than an order of magnitude less than that for TBAH^+^, it appeared to be still higher than that reported from the MD simulations at higher bulk concentrations of pure HAH^+^. When comparing the measured signal intensity ratios with the concentration ratios from the MD simulations, those measured for the HA solutions are lying between the concentration ratios for the solutions with HAH^+^ and HA. Here, we note that quantitative comparison is difficult due to the many assumptions made when applying the attenuation model to retrieve concentrations from the XPS data. These include the following: the assumed layered structure rather than electron scattering calculations with the distinct density profiles obtained by the MD simulations and the fact that we used the average density profiles rather than the instantaneous interface to guide the attenuation model setup;^50^ uncertainties in the KE-dependent asymmetry parameter to calculate the photoemission intensities;^33^ uncertainties in the IMFP; and neglecting the role of elastic scattering.^63,80^

The obvious explanation for the observed behavior of Na^+^ or Br^–^ in the presence of TBAH^+^ (experiment), HAH^+^ (MD simulations), and PS^–^ (experiment and MD simulations) are electrostatic effects. Similar effects have also been reported recently for other anion/cation combinations using XPS.^17^ In the MD simulations, we only considered HAH^+^ (to directly compare with HA), but not TBAH^+^. Previous MD simulations have also demonstrated the pronounced surface affinity of hydrophobic TBAH^+^ cations and ion-pairing with the highly polarizable I^–^ anions.^51^Figure S10 in the Supporting Information displays the RDFs for both ions for PS^–^ and HAH^+^, respectively. They show both strong ion-pairing with both contact ion pairs and solvent-separated ion pairs (SSP). In terms of the close to symmetric behavior of Na^+^ and Br^–^ observed here, Gopakumar et al.^81^ noted that the R–NH_3_^+^–Cl^–^ interaction has a stronger attractive effect for Cl^–^ than a repulsive effect for K^+^.

The presence of HAH^+^ in the NaBr solution leads to notable competition for Br^–^ ions, as apparent from the 50% drop of the NaBr coordination number (CN) (Figure S7) and the higher ability of HAH^+^ to attract surrounding Br^–^ ions than Na^+^ (Figure S11). The differences in number of Na^+^ surrounding Br^–^ among the solutions is related to both the difference in numbers of Na^+^ added to ensure charge neutrality but also to the competing interactions. The drop in the CN also results from the fact that Na^+^ is less present at the surface than the HAH^+^ cation and consequently Br^–^. In PS^–^, the higher CN leads to having more Br^–^ interacting with Na^+^, even though PS^–^ drags Na^+^ toward the interface and Br^–^ is depleted from the interface. This higher CN results from the initial conditions. This solution contains indeed an excess of Na^+^ with respect to Br^–^ to ensure electroneutrality and counterbalance the presence of PS^–^ anions. Additionally, the RDFs between the neutral HA headgroup (NH_2_) and different ions indicate that HA does not strongly attract or repel ions at the interface (Figure S12). However, this subtle effect enhances the interaction between Na^+^ and Br^–^ ions by approximately 30% compared to that in a pure NaBr solution. This enhancement means that Na^+^ ions tend to be in contact with Br^–^ ions, showing a mutual influence between them in the presence of HA. For the HA case, Na^+^ shows only a slight increase in the topmost layers, similar to Br-, since the neutral headgroup does not repel Na^+^ as HAH^+^ does (Table S3).

The addition of NaCl led to a smaller enhancement of bromide and sodium in the presence of HAH^+^ and PS^–^, respectively, from both experiment (for bromide, Figure 2) and theory (Table 1). The density profiles (Figure S4) and their integrated relative form (Figure S6) show that bromide and chloride show very similar behavior. Thus, the reduction of bromide at the interface in the presence of NaCl is due to chloride partly replacing bromide in ion-pairs with HAH^+^. This was also discussed in our previous work for TBAH^+^.^11^ For PS^–^, the effect of dilution with additional sodium must be taken into account. Given the significant excess of chloride used in the experimental solutions and the only about one-third reduction of the experimentally observed bromide enhancement indicates that bromide is preferred as ion-pairing partner. Through the salting out effect discussed above, the amount of surfactant at the interface increases, which compensates for part of the bromide signal loss due to competition with chloride. Tomar et al.^82^ reported a small difference in association constants for Br^–^ and Cl^–^ with ammonium (NH_4_^+^). For PS^–^, chloride seems to be more strongly suppressed than bromide. Zhao et al.^83^ demonstrated that Br^–^ outcompetes Cl^–^ in its attraction to surface tetrahexylammonium (THAH^+^). Ganguly et al.^84^ caution that specific solvent interactions may override ion-pairing effects. Nevertheless, the successful competition of bromide against chloride, as also shown from MD energy distributions (Figures S13 and S14), is likely contributing to the enhanced surface bromide suggested for marine aerosol, despite the low bromide to chloride ratios of 1:650 in bulk seawater.^85^

The present work, of course, remains fairly narrow in scope in that it deals with three individual monofunctional organics and monovalent ions. Especially, divalent ions tend to form stronger ion-pairs and their impact on water structure is more pronounced, which affects, for instance, phase transition behavior, as shown by Unger et al.^86^ The interaction between ions and organics extends far beyond atmospheric chemistry. The way how divalent cations affect the surface propensity of amino acids^87^ or carboxylate and amide groups more generally is relevant in protein denaturing^88^ and all other ion–macromolecule interactions relevant for biochemistry.^89^

## Conclusions

In this study, we use liquid jet XPS to assess the differences among monofunctional surfactants with a positive headgroup (tetrabutyl ammonium), one dominated by the neutral headgroup (HA), and one with a negative headgroup (propyl sulfate) on the abundance of bromide at the interface within the probe depth of XPS. The spectroscopic data were analyzed with the support of an attenuation model to obtain quantitative information about the enhancement of bromide at the interface. Complementary MD calculations were carried out mimicking the mixtures studied experimentally. Both experiments and calculations show that positively charged alkyl ammonium species, along with their surface activity, lead to enhanced interfacial concentration of bromide, while the negatively charged propyl sulfate has only a small effect on the interfacial concentration of bromide. The observed small enhancement of bromide in the presence of the HA at high pH observed by XPS and not confirmed by the MD simulations was likely due to the small fraction of protonated hexylammonium detected at the interface. The MD simulations showed a corresponding enhancement of sodium cations in the presence of propyl sulfate and only minor changes in the presence of neutral HA. In the presence of additional sodium chloride, salting effects and competition for ion-pairing with the ionic surfactant in combination determine the abundance of bromide anions and sodium cations. Therefore, in the presence of soluble surface-active organics, the charge of the headgroup has a substantial impact on the abundance of inorganic ions at the liquid–vapor interface. Thus, to some degree, this conceptual picture allows us to more generally predict how ionic surfactants are affecting the interfacial abundance of the prominent halide, SO_4_^2–^ and NO_3_^–^ anions, and NH_4_^+^ and other cations as a function of pH. However, other effects, such as competition between ion-pairing and molecular interactions among the surfactants, complicate this picture, and more systematic studies are warranted to explore this further.

## Supporting Information Available

Experimental results with additional photoemission spectra and N 1s spectra of reference solutions; description of the attenuation model and detailed results of its application; and supporting theoretical results, with additional density profiles, RDFs, and energy distributions. (PDF)
