# Supplementary material for: Influence of Surfactants with Differently Charged Headgroups on the Surface Propensity of Bromide
Source: J Phys Chem A. 2025 Mar 21;129(13):3085–97. doi: 10.1021/acs.jpca.4c07539 (PMC11973919; doi:10.1021/acs.jpca.4c07539)
Supplement: Supplementary file 1 — jp4c07539_si_001.pdf [file jp4c07539_si_001.pdf]

Supporting Information for Publication

for

Influence of Surfactants with Differently Charged Headgroups on the Surface Propensity of Bromide

Shuzhen Chen[1,2], Rawan Abouhaidar[3], Luca Artiglia[1], Huanyu Yang[1,2], Anthony Boucly[1], Lucia Iezzi[1,2], Jérôme Philippe Gabathuler[1], Thorsten Bartels-Rausch[1], Céline Toubin[3], and Markus Ammann[1*]

1. PSI Center for Energy and Environmental Sciences, Paul Scherrer Institut, 5232 Villigen, Switzerland
2. Department of Environmental System Science, ETH Zurich, 8093 Zürich, Switzerland
3. Univ. Lille, CNRS, UMR 8523 - PhLAM - Physique des Lasers Atomes et Molécules, F-59000 Lille, France

Corresponding Author
*markus.ammann@psi.ch (M. Ammann)

Section S1. Supporting experimental results

C 1s, N 1s, S 2p, and Cl 2p spectra taken at KE=155 eV

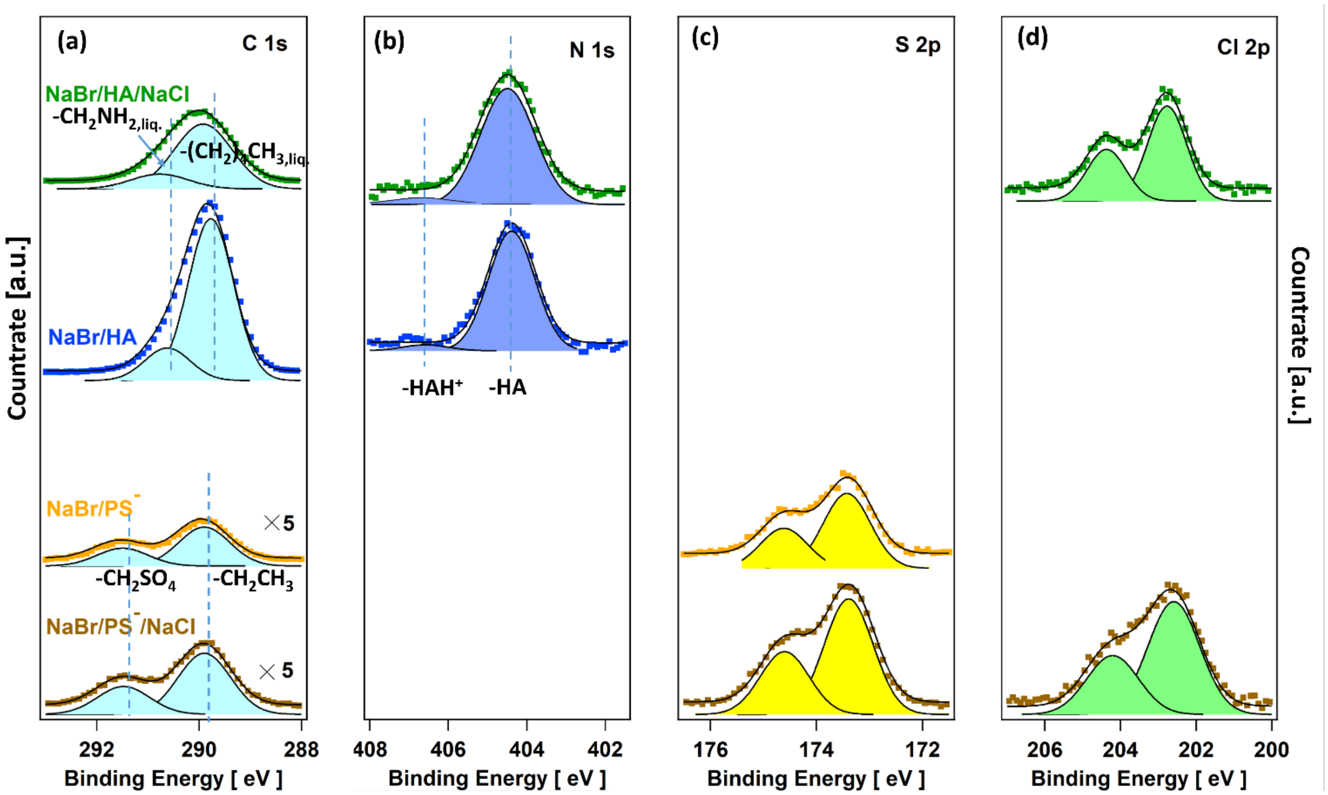

Figure S1: a) C 1s, b) N 1s, c) S 2p, and d) Cl 2p spectra of mixed 0.1 M NaBr/0.1 M hexylamine (HA), mixed 0.1 M NaBr/0.1 M hexylamine/0.55 M NaCl, mixed 0.1 M NaBr/0.1 M sodium propylsulfate (PS^-) and mixed 0.1 M NaBr/0.1 M sodium propylsulfate/0.55 M NaCl aqueous solutions, which were measured at photon energies of 448 eV for C 1s, 560 eV for N 1s, 330 eV for S 2p and 360 eV for Cl 2p to obtain the same kinetic energy of around 155 eV.

The C 1s (Figure S1a) exhibits two features representing aliphatic chain carbon ($-C_5H_{11}$) and the amine coupled carbon ($-CH_2-NH_2$) at a binding energy of 289.7 and 290.5 eV, respectively, for solutions in the presence of hexylamine. Correspondingly, the two features represent aliphatic chain carbon ($-C_2H_5$) and the sulfate coupled carbon ($-CH_2-OSO_3^-$) at a binding energy of 289.7 and 291.3 eV, respectively, for solutions in the presence of propyl sulfate. Note that the C 1s spectra for propyl sulfate in Figure S1a are enlarged by a factor of 5. The lower C 1s intensity for the propyl sulfate is due to the lower surface activity of this solute and the shorter aliphatic carbon chain.

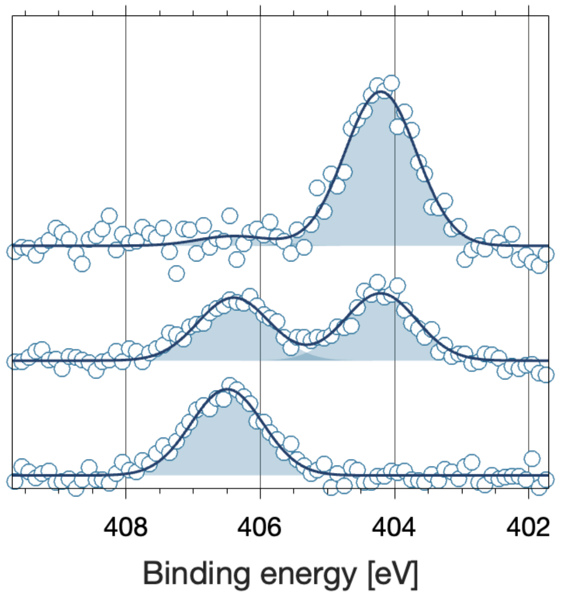

Figure S2: N 1s spectra (open circles) of mixed 0.1 M hexylamine solutions at pH 12.2 (top), pH 10.2 (middle) and pH 8.2 (bottom). The incident photon energy was 570 eV.

Reference spectra of HA solutions at pH below, equal to the pKa and above that are shown in Figure S2. N 1s spectra (open circles) of mixed 0.1 M hexylamine solutions at pH 12.2 (top), pH 10.2 (middle) and pH 8.2 (bottom). The pH was set at room temperature by adding NaOH or H_2SO_4 to the hexylamine solutions. The incident photon energy was 570 eV. The binding energy was scaled relative to the vacuum level using the C-H feature at 289.7 eV of C1s spectra as a reference. The higher BE peak is hexyl ammonium. Fitting the three data sets simultaneously with 2 Gaussians each, constrained to the same binding energy for hexylamine at 403.5-404.eV and an energy separation of 1.5-3.0 eV for both features in each data set, and the same FWHM each between 1-3 eV, yields a peak separation of 2.2 eV, a FWHM of 1.25 eV, and a peak area for hexyl ammonium of 0.057 and 0.90 for hexylamine at pH 12.2. BE, separation between hexyl ammonium and amine is similar to previous work (1, 2). The sum of the Gaussians (shaded area) represents the data well (blue line). Thus, the apparent degree of protonation at pH 12.2 was 6 %, which is also comparable to previous measurements at the same pH.(3) The N 1s region in Figure S1b, thus the mixtures with NaBr and additional NaCl, was then fitted by taking the fit of the reference spectra as a constraint, yielding 5%. The pH of these solutions was around 12, measured at 294 K, while the pKa of hexyl ammonium (HAH^+) is 10.64 at 298 K.(4) As explained in the methods section of the main text, we estimate the temperature being at around 283 K. The pKa of HAH^+ increases from 10.6 at 298 K to 11.2 at 283 K.(5) In turn, the pH of the 0.1 M HA solution used for the mixture with 0.1

M NaBr is calculated to be at 11.8 at 298 K and 12.1 at 283 K through the autodissociation equilibrium (the pH was not adjusted). Therefore, the increase of the pKa at lower temperature should lead to a fraction of protonated HAH^+ of 11 % in the bulk, which is above what was measured in N1s spectra of the mixed solution and also more than that in the reference solution at pH 12.2. This is in line with the higher surface propensity of the neutral hexylamine than that of the charged hexylammonium, as discussed in the main text.

The S 2p spectra exhibit the spin-orbit split S $2p^{3/2}$ and $2p^{1/2}$ peaks (Figure S1c) at a binding energy of 173.5 eV and 174.7 eV, respectively, with a spin-orbit split of 1.2 eV for sulfate. The Cl 2p spectra (Figure S1d) show the spin-orbit split contributions of Cl $2p^{3/2}$ and $2p^{1/2}$ at a binding energy of 202.8 eV and 204.4 eV, respectively, and a spin-orbit split of 1.4 eV for chloride.

Upon adding NaCl, the C 1s signal intensity decreases for the hexylamine solutions, but increases for the propyl sulfate solutions. The S 2p signal for the propyl sulfate solutions responds in a similar way to the presence of NaCl. The Cl 2p signal is slightly higher in NaBr/propyl sulfate/NaCl than in NaBr/HA/NaCl solutions due to less attenuation by the aliphatic carbon layer at the surface in presence of propyl sulfate. Similar to the Br 3d and O 1s spectra, the peaks widen in presence of NaCl.

Section S2. Calculation of photoemission signal intensity ratios using the photoelectron attenuation model to obtain interfacial enhancements of bromide

For facilitating the comparison of the integrated density profiles for bromide derived from the MD simulations with the XPS results, we applied a simple attenuation model developed previously.(6, 7) It is used to calculate photoemission signals as a function of kinetic energy and to fit to the data in Figure 2. It is based on an assumed density profile and that takes into account attenuation of photoelectrons originating from different depths. The model setup of the interfacial region is provided in Figure S3. We assume that the aliphatic carbon chains reside above (on the gas side of) the interface and that the headgroup ($–NH_2$, $-NH_3^+$ or $-OSO_3^-$) is residing just at the interface. We do not explicitly resolve that these headgroups, being likely hydrated, reside slightly below the water surface. We further consider a region of thickness Δ within which the bromide concentration differs from that in the bulk aqueous phase, as in Lee et al..(6) This simple representation of the interface allows an analytical solution to calculate the photoemission signals. In principle, the detailed density profiles as returned by the MD simulations could be used to predict the photoemission signals based on detailed electron scattering calculations.(8) However, this was beyond the scope of this work, since, as already mentioned above, the equilibrated concentrations within the simulation box did not match those of the experiment to warrant a more detailed calculation.

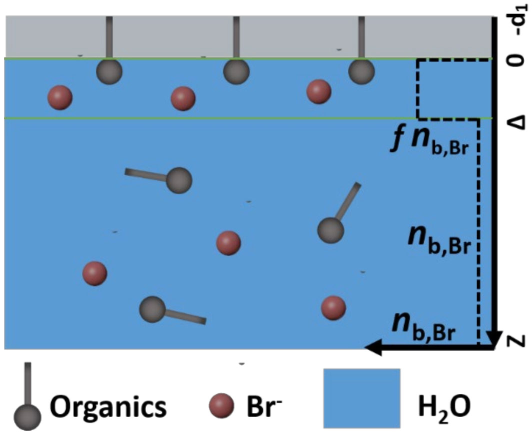

Figure S3: Scheme of the organic compounds covering the interface used for the attenuation model. The reference level 0 for the depth scale (z) is where the water density drops (assuming a rectangular profile). The aliphatic carbon chains reside above that, at $z < 0$, while the layer following next, with thickness Δ, represents the layer in which the concentration of bromide is deviating from its bulk value, $n_{b,Br}$, by the factor *f*. The bromide density profile is schematically depicted as the dashed line. Red and light grey

spheres denote bromide ions and headgroup (in our case, $–NH_2$ or $–OSO_3^-$) of organic compounds, respectively.

The layer of thickness Δ, just below the aliphatic carbon layer, starts at depth $z = 0$, within the aqueous solution. In this layer, the Br atom density is set different from that of the bulk density by a factor, f, i.e., $fn_{\mathrm{b,Br}}$, while the density of the organic (within the layer) is equal to that in the bulk. We choose the same interfacial thickness as for the integration of the density profiles obtained from the MD simulations, Δ = 0.5 nm. f is a function of the surface excess of the surfactant, Γ_{hex} or Γ_{org}. In the absence of organic $f = f_0$ is related to the surface excess of the bromide ions, $\Gamma_{\mathrm{Br-}}$, via:

$$\Gamma_{Br-} = n_{\mathrm{b,Br}}\Delta(f_0 - 1) \qquad f_0 = \frac{\Gamma_{Br-} - n_{\mathrm{b,Br}}\Delta}{-n_{\mathrm{b,Br}}\Delta} = \frac{\Gamma_{Br-}}{n_{\mathrm{b,Br}}\Delta} + 1 \qquad \text{(S1)}$$

Since we have not re-assessed the depth profile of Na and Br with independent, calibrated KE dependent experiments, we take f_0 = 0.1, close to that of 0.06 from the simulations (see Table S3 below). This depletion corresponds to a negative surface excess of bromide of -2.7×10^{12} cm^{-2} (Equation S1), which is close to the surface excess derived from the measured surface tension, -2.6×10^{12} cm^{-2},(9) which we used in our previous work.(6, 7) This leads to an effective surface concentration of 3×10^{11} ions cm^{-2} (see Table S1), roughly consistent with the value obtained from the MD simulations reported in Table S3 when taking into account the difference in bulk concentration. The choice of f_0 = 0.1 and Δ = 0.5 nm, different to that used in our previous work, was used to allow the comparison with the MD simulations. The true density increase is not vertical, as obvious from the MD simulations. Therefore, the contribution of atoms contained within this layer to the photoemission signal calculated below is obviously sensitive to this choice. However, we note that the depletion assumed here of 0.1 over 0.5 nm depth is roughly consistent with an average depletion of 0.5 over a depth of 1.0 nm.

In the presence of surfactants, the aliphatic portion of tetrabutylammonium, hexylamine or propylsulfate resides above the aqueous phase. In this work, we refrain from quantifying the effective thickness of this layer as done previously.(6, 7) Instead, we assume that $H_2O(l)$, as well as Na^+ and Br^- are always residing below this layer, thus neglecting the effect of the surfactants on the density profile of water returned by the MD simulations. In the calculation of the photoemission signals, Br 3d and O 1s photoelectrons are assumed to be all attenuated by the same factor, $e^{-\mathrm{d1}/\lambda}$, and thus, the resulting Br/O ratios are not depending on the thickness of this layer.

The photoemission signal from O atoms, assumed to be homogeneously distributed over the bulk up to below the aliphatic carbon layer, which is not containing oxygen, is given by:

$$I_{\mathrm{O1s,\,bulk}} = Ae^{-\mathrm{d1}/\lambda}\int_0^\infty n_{\mathrm{b,O}}e^{-(z)/\lambda}dz = Ae^{-\mathrm{d1}/\lambda}n_{\mathrm{b,O}}\lambda \qquad \text{(S2)}$$

λ is the electron inelastic scattering mean free path. We used $\lambda = 2/pi*\lambda_0$ to account for the liquid jet measurement configuration.(10) λ_0 is the inelastic mean free path for electrons in water.(11) The factor *A* convolutes the measurement efficiency depending on cross section, photon flux and analyzer transmission.

In absence of organics, the Br photoemission signal intensity can be obtained as follows:

$$I_{\text{Br-}} = Be^{-\text{d}1/\lambda}\left(\int_0^{\Delta} f_0 n_{\text{b,Br-}} e^{-\frac{(z)}{\lambda}} dz + e^{-\frac{\Delta}{\lambda}} \int_{\Delta}^{\infty} n_{\text{b,Br-}} e^{-\frac{(z-\Delta)}{\lambda}} dz\right)$$

$$= Be^{-\text{d}1/\lambda}\lambda f_0 n_{\text{b,Br-}}(1-e^{-\frac{(z)}{\lambda}}) + Be^{-\text{d}1/\lambda}\lambda n_{\text{b,Br-}} e^{-\frac{\Delta}{\lambda}} \quad \text{(S3)}$$

For 0.1 M NaBr and 0.1 M NaBr/0.55 M NaCl solutions, the Br 3d to O 1s signal intensity ratio is:

$$\frac{I_{\text{Br-}}}{I_{\text{O1s}}} = \frac{B\lambda f_0 n_{\text{b,Br-}}(1-e^{-\frac{(z)}{\lambda}}) + B\lambda n_{\text{b,Br-}} e^{-\frac{\Delta}{\lambda}}}{A n_{\text{b,O}}\lambda} = \frac{\text{B}}{\text{A}} \frac{f_0 n_{\text{b,Br-}}(1-e^{-\frac{(z)}{\lambda}}) + n_{\text{b,Br-}} e^{-\frac{\Delta}{\lambda}}}{n_{\text{b,O}}} \quad \text{(S4)}$$

If the organic surfactant is present in the solutions, the Br photoemission signal intensity is obtained from the following integration:

$$I_{\text{Br}} = Be^{-\frac{d1}{\lambda}}\left[\int_0^{\Delta} f n_{\text{b,Br}} e^{-(z)/\lambda} dz + e^{-\frac{\Delta}{\lambda}} \int_{\Delta}^{\infty} n_{\text{b,Br}} e^{-(z-\Delta)/\lambda} dz\right] =$$

$$I_{\text{Br}} = Be^{-\frac{d1}{\lambda}} \left[f n_{b,Br}\lambda - f n_{b,Br}\lambda e^{-\Delta/\lambda} + n_{b,Br}\lambda e^{-\frac{\Delta}{\lambda}}\right]$$

$$I_{\text{Br}} = Be^{-\text{d}1/\lambda} n_{b,Br}\lambda\left[f + e^{-\Delta/\lambda}(1-f)\right] \quad \text{(S5)}$$

Then, the Br 3d to O 1s signal intensity ratio is obtained from equation S2 and equation S5 (the attenuation factor due to the aliphatic overlayer cancels out):

$$\frac{I_{\text{Br}}}{I_{\text{O1s}}} = \frac{Be^{-\text{d}1/\lambda} n_{b,Br}\lambda\left[f+e^{-\Delta/\lambda}(1-f)\right]}{A n_{\text{b,O}}\lambda e^{-d1/\lambda}} = \frac{B n_{b,Br}\left[f+e^{-\Delta/\lambda}(1-f)\right]}{A n_{\text{b,O}}} \quad \text{(S6)}$$

When we normalize the Br 3d to O 1s signal intensity ratio in presence of the organic solutes (equation S6) to that of the neat 0.1 M NaBr solution (equation S4), the calibration factors *A* and *B* cancel out:

$$\frac{\frac{I_{\text{Br-}}}{I_{\text{O1s}}}, org}{\frac{I_{\text{Br-}}}{I_{\text{O1s}}}, NaBr} = \frac{\frac{B n_{b,Br}\left[f+e^{-\Delta/\lambda}(1-f)\right]}{A n_{\text{b,O}}}}{\frac{B n_{b,Br}\left[f_0+e^{-\Delta/\lambda}(1-f_0)\right]}{A n_{\text{b,O}}}} = \frac{\left[f+e^{-\Delta/\lambda}(1-f)\right]}{f_0+e^{-\Delta/\lambda}(1-f_0)} \quad \text{(S7)}$$

We then determine *f* by fitting equation (S7) to the measured Br 3d to O 1s signal ratios for the solutions. Note that *f* is the only variable for this fit. The fitted intensity ratios are shown as lines in Figure 2 of the main text. The values obtained from these fits are reported in Table S1 below and summarized in Table 1 of the main text. To assess the uncertainty for the fitted values of *f* presented in Table S1, we have varied f_0 from 0.06 to 0.3 and Δ between 0.5 and 1.0 nm.

Table S1: Calculated interfacial concentrations of bromide and the driving parameters were obtained for the different solutions. The error associated with the individual photoemission signal intensities is estimated at around ±10%, which considers uncertainties due to the variability of the liquid-jet XPS experiment, the solution preparation and spectral fitting. Propagation leads to around 15% for elemental ratios and around 20% for the concentrations given. However, the overall uncertainty is dominated by the choice of the value for Δ and f_0, as described in the text.

Solutions	$n_{Br,b}$ [M]	f_{Br}	Error (f_{Br})	$n_{Br,\Delta}$ [M]	$\Delta \times n_{Br,\Delta}$ [10^{13} cm^{-2}]	[$n_{Br,\Delta}$/ $n_{Br,b}$]$_{norm}$ (exp)
0.1 M NaBr	0.1	0.1	/	0.01	0.03	1.0
0.1 M NaBr/0.1 M Hexylamine	0.1	2.0	0.27	0.20	0.60	20.0
0.1 M NaBr/0.1 M Hexylamine/0.55 M NaCl	0.1	1.3	0.20	0.13	0.39	12.9
0.1 M NaBr/0.1 M Propylsulfate	0.1	0.003	0.07	0.00	0.00	0.03
0.1 M NaBr/0.1 M Propylsulfate/0.55 M NaCl	0.1	0.2	0.08	0.02	0.07	2.2
0.1 M TBA-Br	0.1	35.2	1.74	3.5	10.59	351.8
0.1 M TBA-Br/0.55 M NaCl	0.1	16.8	0.42	1.7	5.07	168.4

Section S3. Supporting theoretical results: Classical MD

Table S2: RESP Charges (B3LYP/6-311+G(d)) utilized in each simulation for the various organic molecules

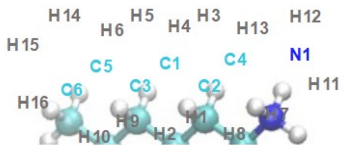

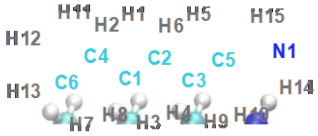

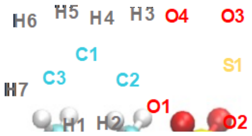

Hexylammonium			Hexylamine			Propylsulfate		
Number	Atom	Partial Charge	Number	Atom	Partial Charge	Number	Atom	Partial Charge
1	N1	-0.319945	1	C6	-0.102400	1	C6	-0.102400
2	H11	0.313693	2	H11	0.018185	2	H11	0.018185
3	H12	0.313693	3	H12	0.018185	3	H12	0.018185
4	H13	0.313693	4	H13	0.018185	4	H13	0.018185
5	C4	0.035956	5	C4	0.072746	5	C4	0.072746
6	H7	0.094390	6	H7	-0.008645	6	H7	-0.008645
7	H8	0.094390	7	H8	-0.008645	7	H8	-0.008645
8	C2	-0.018230	8	C1	0.049246	8	C1	0.049246
9	H3	0.031738	9	H1	-0.014557	9	H1	-0.014557
10	H4	0.031738	10	H2	-0.014557	10	H2	-0.014557
11	C1	-0.009845	11	C2	-0.042464	11	C2	-0.042464
12	H1	0.016656	12	H3	-0.014504	12	H3	-0.014504
13	H2	0.016656	13	H4	-0.014504	13	H4	-0.014504
14	C3	-0.016756	14	C3	0.039114	14	C3	0.039114
15	H5	0.019345	15	H5	0.014313	15	H5	0.014313
16	H6	0.019345	16	H6	0.014313	16	H6	0.014313
17	C5	0.022685	17	C5	0.263483	17	C5	0.263483
18	H9	0.019081	18	H9	-0.001559	18	H9	-0.001559
19	H10	0.019081	19	H10	-0.001559	19	H10	-0.001559
20	C6	-0.115091	20	N1	-1.030067	20	N1	-1.030067
21	H14	0.039242	21	H14	0.372845	21	H14	0.372845
22	H15	0.039242	22	H15	0.372845	22	H15	0.372845
23	H16	0.039242	-	-	-	-	-	-

Table S3: Number of ions and water molecules used for each mixture; characteristic results obtained from integrating the density profiles after equilibration**.**

	Starting composition of slab (number of ions / molecules)					Data compiled from the density profile integration after equilibration																
						Interfacial concentration (within 0.5 nm) in M (average of 1 and 2)				Interfacial concentration in ion or molecule / cm^2 (within 0.5 nm)				Bulk concentration in M				Ratio Br interface to bulk (M/M)	Norm. ratio Br	Ratio Na interface to bulk (M/M)	Norm. ratio Na	Ratio Org. interface to bulk (M/M)
	Na^+	Br^-	Cl^-	Org.	H_2O	Na^+	Br^-	Cl^-	Org.	Na^+	Br^-	Cl^-	Org.	Na^+	Br^-	Cl^-	Org.	$[Br]_i/[Br]_b$		$[Na]_i/[Na]_b$		$[Org.]_i/[Org.]_b$
**NaBr solution**																						
1M NaBr	50	50	-	-	2775	0.06 ± 0.01	0.06 ± 0.01	-	-	1.85E+12	1.95E+12	-	-	1.09	1.09	-	-	0.06 ± 0.01	1	0.06 ± 0.01	1	-
1 M NaBr + 1M NaCl	100	50	50	-	2775	0.17 ± 0.01	0.11 ± 0.02	0.07 ± 0.01	-	5.00E+12	3.22E+12	2.24E+12	-	2.17	1.08	1.09	-	0.10 ± 0.02	1.66	0.08 ± 0.01	1.35	-
**Hexylammonium solution**																						
0.5M NaBr + 0.5M HexylammoniumBr	25	50	-	25	2775	0.02 ± 0.00	0.29 ± 0.01	-	0.91 ± 0.02	6.06E+11	8.85E+12	-	2.74E+13	0.55	1.03	-	0.31	0.28 ± 0.01	4.77	0.04 ± 0.01	0.65	2.90 ± 0.02
0.5M NaBr + 0.5M HexylammoniumBr + 1M NaCl	75	50	50	25	2775	0.07 ± 0.02	0.28 ± 0.01	0.23 ± 0.01	1.18 ± 0.07	2.17E+12	8.38E+12	6.88E+12	3.56E+13	1.66	1.04	1.06	0.24 ± 0.02	0.27 ± 0.02	4.47	0.04 ± 0.02	0.77	4.99 ± 0.10
**Hexylamine solution**																						
1M NaBr + 0.5M Hexylamine	50	50	-	25	2775	0.09 ± 0.01	0.09 ± 0.01	-	1.89 ± 0.02	2.60E+12	2.63E+12	-	5.68E+13	1.12	1.12	-	0.05	0.08 ± 0.01	1.31	0.08 ± 0.02	1.37	-
1M NaBr + 0.5M Hexylamine + 1M NaCl	100	50	50	25	2775	0.17 ± 0.04	0.10 ± 0.02	0.07 ± 0.02	1.87 ± 0.02	5.00E+12	2.98E+12	2.18E+12	5.63E+13	2.19 ± 0.01	1.09 ± 0.01	1.10 ± 0.01	0.05	0.09 ± 0.03	1.52	0.08 ± 0.05	1.34	-
**Propylsulfate solution**																						
1M NaBr + 0.5M PropylsulfateNa	75	50	-	25	2775	0.23 ± 0.01	0.06 ± 0.01	-	0.61 ± 0.05	6.96E+12	1.76E+12	-	1.82E+13	1.57	1.07	-	0.39 ± 0.01	0.05 ± 0.01	0.91	0.15 ± 0.01	2.60	1.57 ± 0.06
1M NaBr + 0.5M PropylsulfateNa + 1M NaCl	125	50	50	25	2775	0.38 ± 0.03	0.08 ± 0.02	0.05 ± 0.02	0.95 ± 0.07	1.15E+13	2.31E+12	1.50E+12	2.86E+13	2.63 ± 0.01	1.07 ± 0.01	1.08 ± 0.01	0.29 ± 0.02	0.07 ± 0.03	1.20	0.15 ± 0.03	2.57	3.23 ± 0.09

Table S4: Interfacial and bulk volumes used to calculate concentrations

	L_x (nm)	L_y (nm)	**Interfacial region = 10% to 90% of water density**				Interface thickness (nm)	**Interfacial volume (nm^3)**	Bulk thickness (nm)	**Bulk volume (nm^3)**
			Interface 1 (nm)		Interface 2 (nm)					
**NaBr solution**										
1M NaBr	4.39	4.39	4.75	5.25	9.15	9.65	0.5	9.64	3.9	75.16
1 M NaBr + 1M NaCl	4.43	4.43	4.75	5.25	9.07	9.57	0.5	9.81	3.82	74.96
**Hexylammonium solution**										
0.5M NaBr + 0.5M HexylammoniumBr	4.46	4.46	4.8	5.3	9.05	9.55	0.5	9.96	3.75	74.71
0.5M NaBr + 0.5M HexylammoniumBr + 1M NaCl	4.51	4.51	4.9	5.4	9.05	9.55	0.5	10.15	3.65	74.08
**Hexylamine solution**										
1M NaBr + 0.5M Hexylamine	4.47	4.47	5.15	5.65	9.3	9.8	0.5	9.98	3.65	72.87
1M NaBr + 0.5M Hexylamine + 1M NaCl	4.51	4.51	4.9	5.4	9.05	9.55	0.5	10.17	3.65	74.25
**Propylsulfate solution**										
1M NaBr + 0.5M PropylsulfateNa	4.45	4.45	4.75	5.25	9.1	9.6	0.5	9.92	3.85	76.37
1M NaBr + 0.5M PropylsulfateNa + 1M NaCl	4.49	4.49	4.8	5.3	9.07	9.57	0.5	10.08	3.77	75.99

Density profiles in presence of NaCl

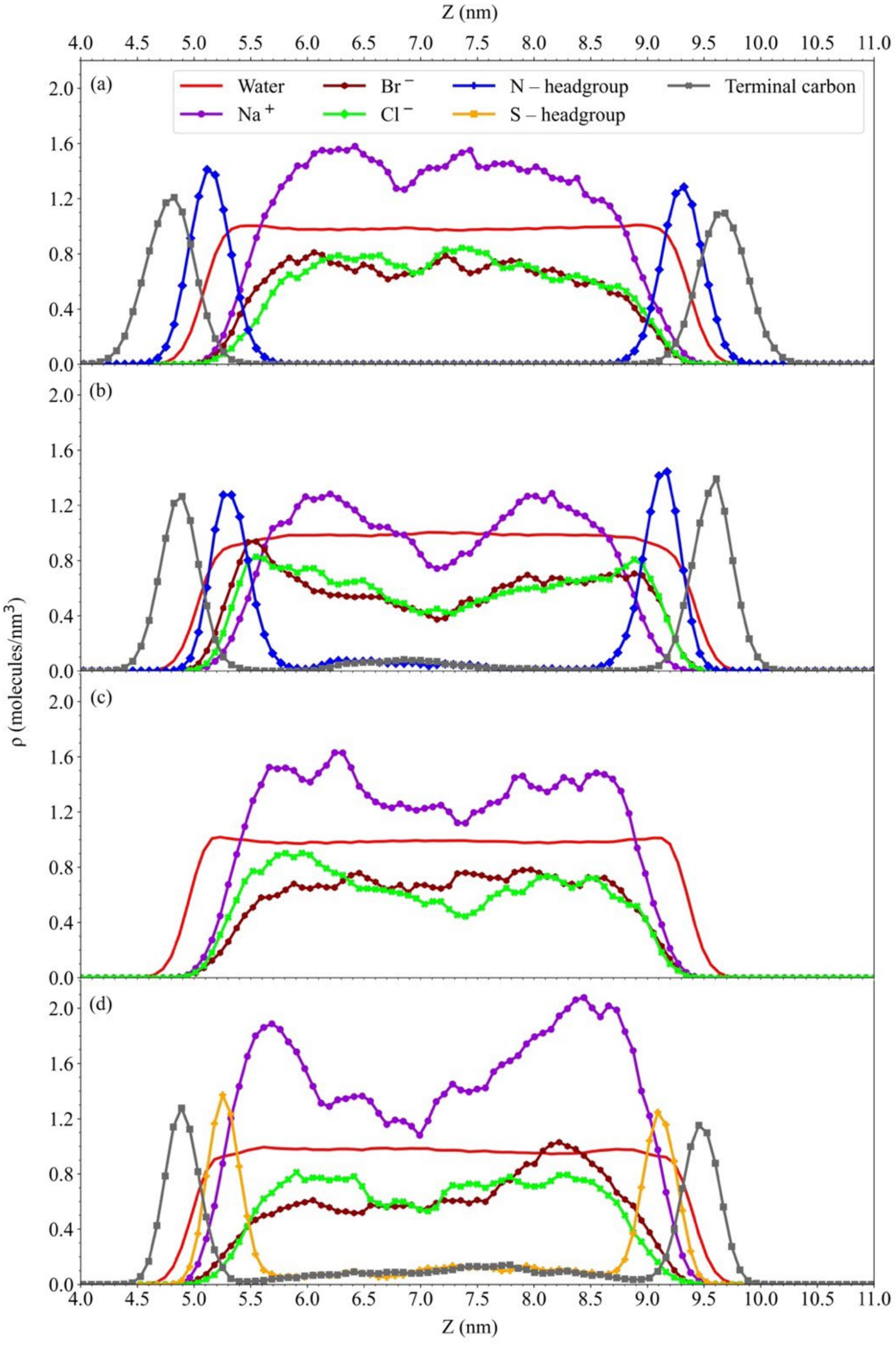

Figure S4: Density profiles of (a) mixed 1M NaBr/0.5M hexylamine/1M NaCl, (b) mixed 0.5M NaBr/0.5M hexyl ammonium bromide/1M NaCl, (c) 1M NaBr/1M NaCl, and (d) mixed 1M NaBr/0.5M sodium propyl sulfate/1M NaCl aqueous solutions. Densities of water molecules are shown in red, Na^+ ions in violet, Br^- ions in dark red, Cl^- ions in green, terminal carbon chains in gray, and the headgroups of hexyl ammonium, hexylamine, and propyl sulfate are in dark blue, and yellow respectively. To seek clarity, water density has been scaled so that we can more easily identify the bulk and interface regions.

Relative density profiles

For Figure S5 and S6 densities were integrated throughout the slab in the z-direction with an interval distance of 0.5 nm. Through this integration, we can calculate the ratio of a given ion over a certain z interval with respect to bulk, describing the surface propensity of each ion in a normalized manner in the presence of the different studied organic compounds. We focus on the same 0.5 nm interfacial region chosen before where z is comprised approximately between 9 and 9.5 nm for the upper interface (grey shadings in Figures S5 and S6). This reiterates the clear interfacial enhancement of Br^- in presence of HAH^+ and of Na^+ in presence of PS^-, but with higher resolution. In Figure S6, we observe that the addition of NaCl maintains the same trend, with Cl^- being more present at the surface in the presence of HAH^+.

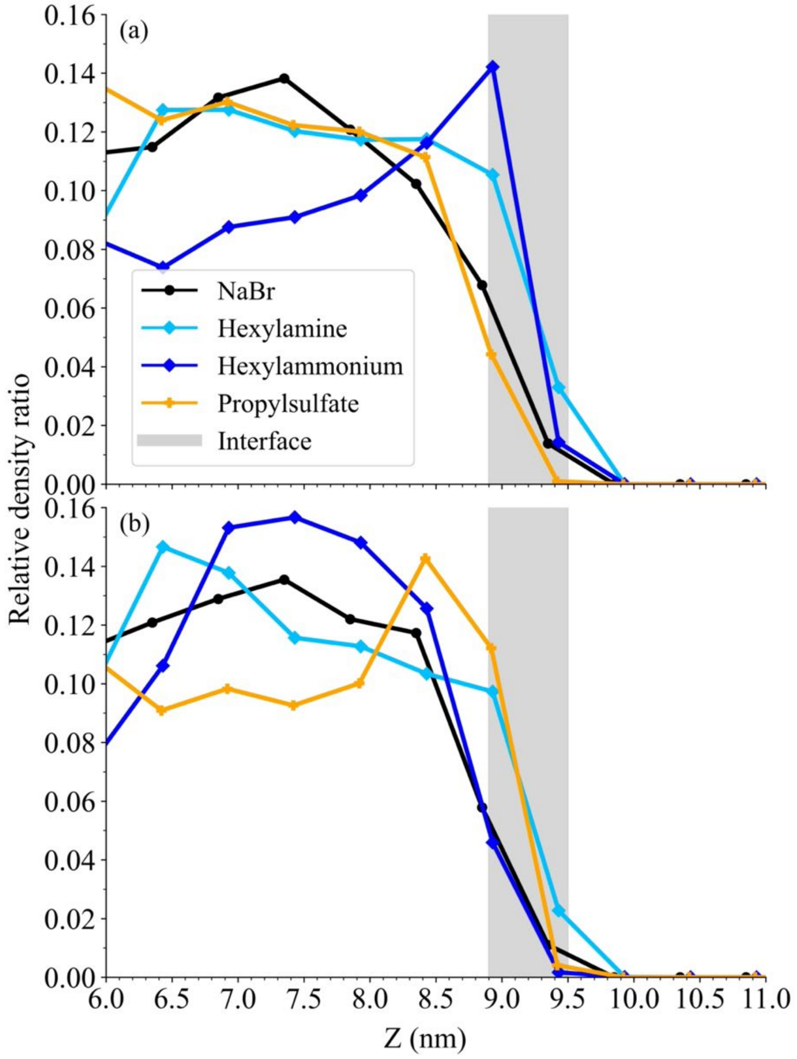

Figure S5: Relative density ratios of (a) Br^- and (b) Na^+ ions within various solutions. Density integration was conducted along the z-axis at 0.5 nm intervals throughout the slab. Interfacial region is highlighted in grey shading.

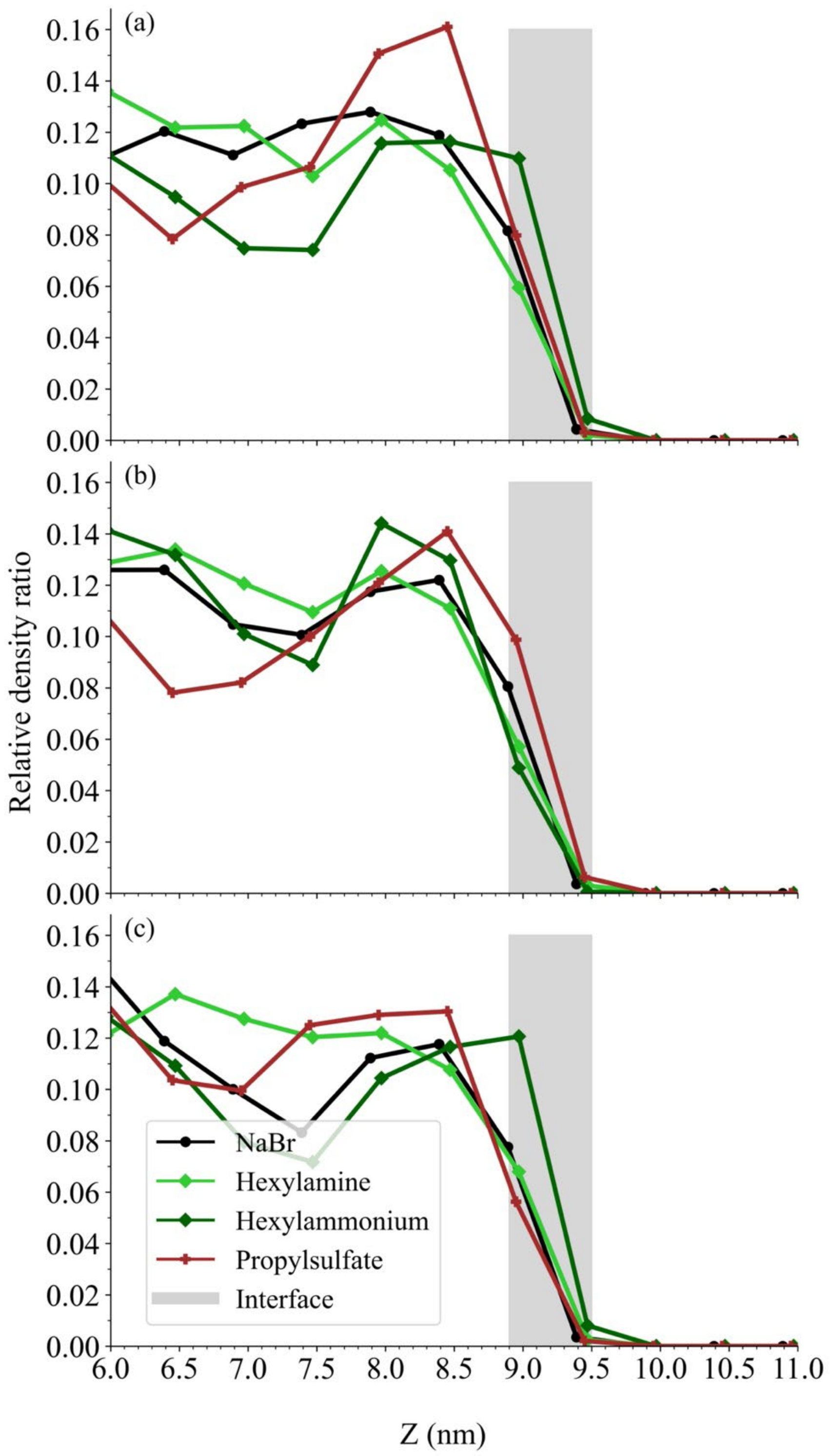

Figure S6: Relative density ratios of (a) Br^-, (b) Na^+ and Cl^- ions within various solutions with the addition of NaCl. Density integration was conducted along the z-axis at 0.5 nm intervals throughout the slab. Interfacial region is highlighted in grey shading.

Radial distribution functions and coordination numbers

Radial distribution functions among ions and between surfactant ions and water are shown in Figures S7 and S8. The nearly identical density profiles of Na^+ and Br^- are due to ion-ion interactions. This is also apparent from the RDF between Na^+ -- Br^- pairs provided in Figure S8. The first peak at 0.29 nm represents the Contact Ion Pair (CIP), showing direct contact between ions without any solvent molecules in between. This indicates a strong ion-ion attraction. The second peak, occurring around 0.5nm, corresponds to the Solvent Separated Ion Pair (SSIP).(12) Here, Na^+ and Br^- ions are separated by one or more solvent molecules, reducing their direct interaction. These distances for the CIP and SSIP shells are consistent with observations from previous studies.(13, 14) By integrating the RDF, the figure also provides Coordination Numbers (CN), indicating the number of Na^+ ions around each Br^- ion. In the first solvation shell, the approximately half distribution suggests a dynamic nature involving a varying number of solvent molecules between the ions throughout the aqueous solutions. The remaining Figures S9 – S15 provide further radial distribution function and coordination numbers for the other solute ions, as well as the energy distributions for the different ion pairs discussed in the main text.

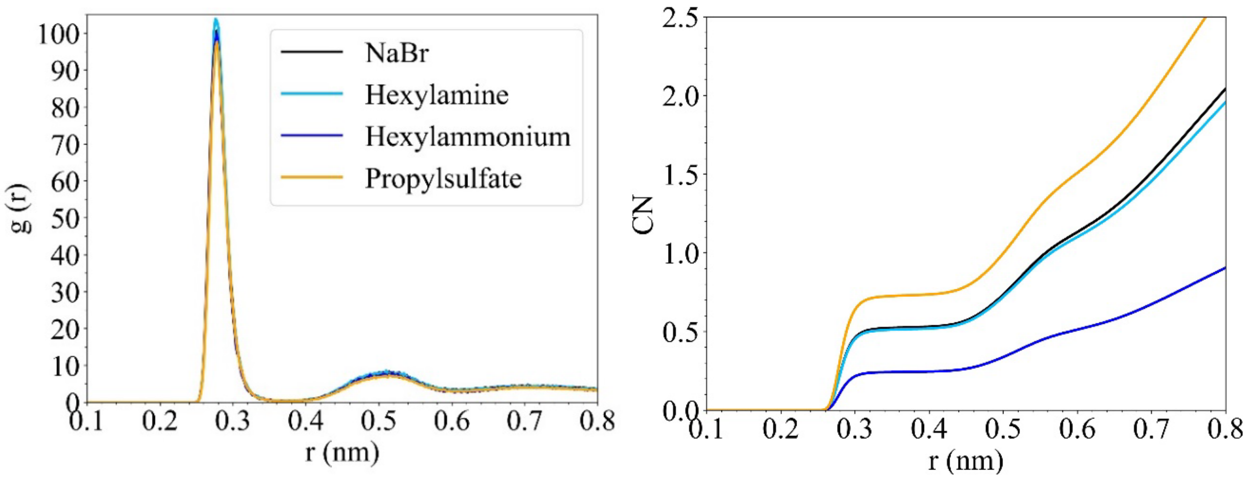

Figure S7: Radial distribution function and coordination number between Na^+ and Br^- pairs in different solutions: pure NaBr, with HA, with HAH^+, and with PS. The initial peak represents the Contact Ion Pair (CIP), while subsequent peaks correspond to Solvent Separated Ion Pairs (SSIP).

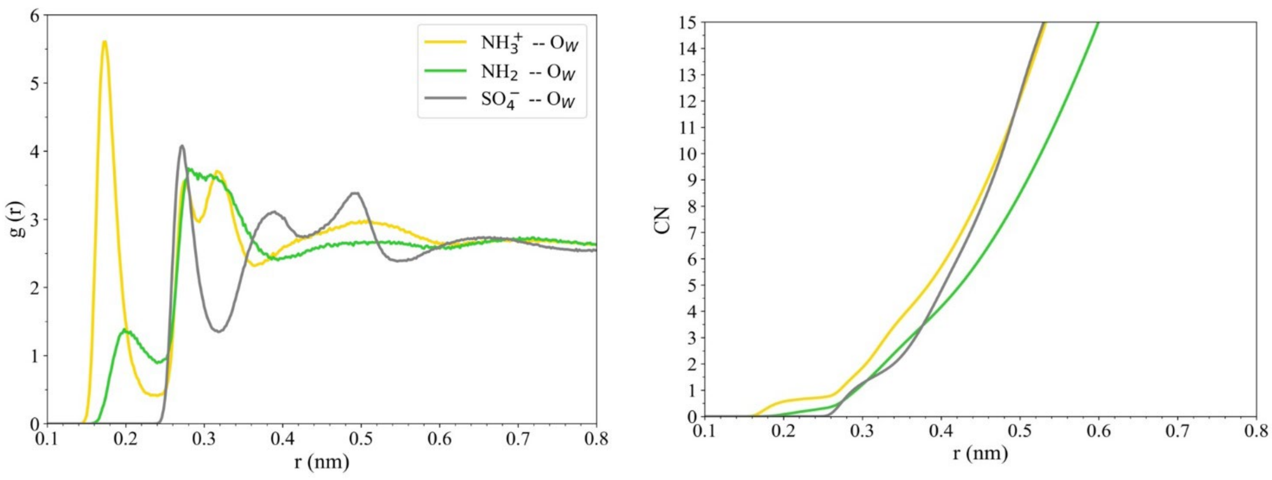

Figure S8: Comparative (top) radial distribution functions and (bottom) coordination numbers elucidating the interactions of organic solute headgroups in an aqueous environment. NH_3^+, NH_2, SO_4- denote the headgroup of hexylammonium, hexylamine, and propylsulfate respectively. O_w refers to oxygen atoms within water molecules.

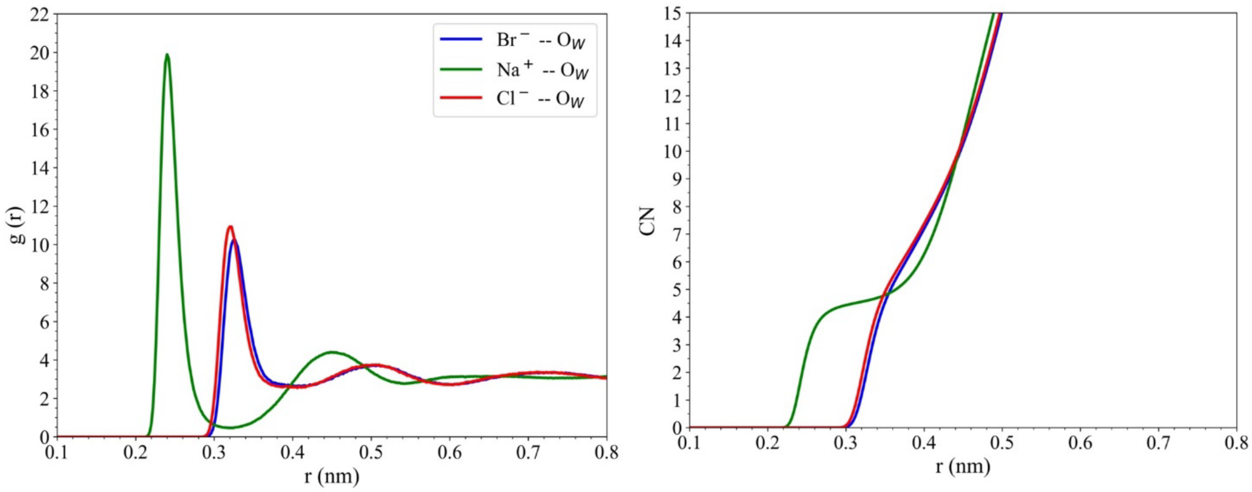

Figure S9: Comparative (a) radial distribution functions and (b) coordination numbers elucidating the interactions of different ions (Br^-, Na^+, and Cl^-) in an NaBr/NaCl aqueous environment. O_w refers to oxygen atoms within water molecules.

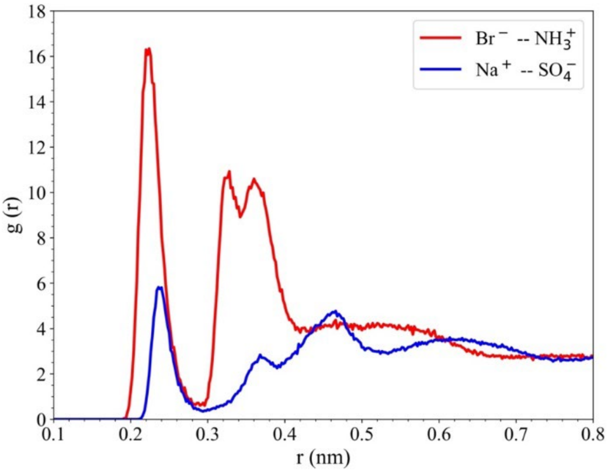

Figure S10: Radial distribution functions of the electrostatic interactions between ionic species: sodium with propylsulfate, and hexylammonium with bromide.

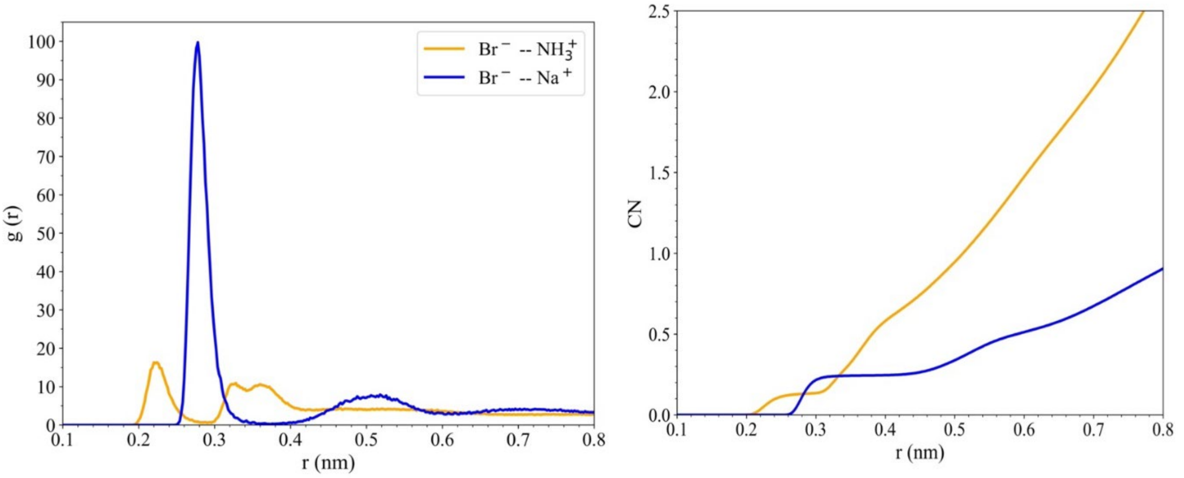

Figure S11: Analysis of radial distribution function and coordination number between Br^- ions with Na^+, and hexylammonium headgroups (NH_3^+) in a 0.5M NaBr/0.5M hexylammonium bromide aqueous solution.

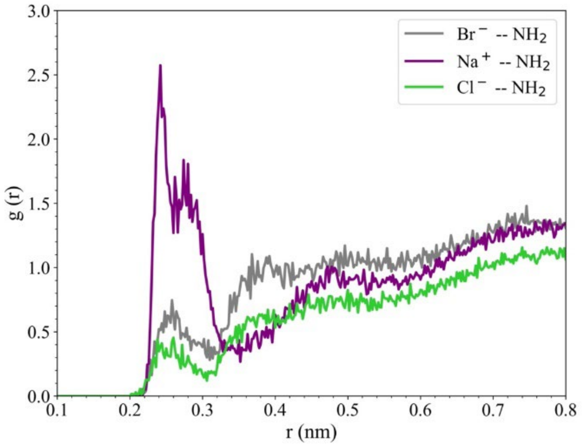

Figure S12: Radial distribution functions between the neutral hexylamine headgroup (NH_2) with the various ions (Na^+, Br^-, and Cl^-) in mixed 1M NaBr/0.5M hexylamine/1M NaCl solution.

Competition between bromide and chloride ions

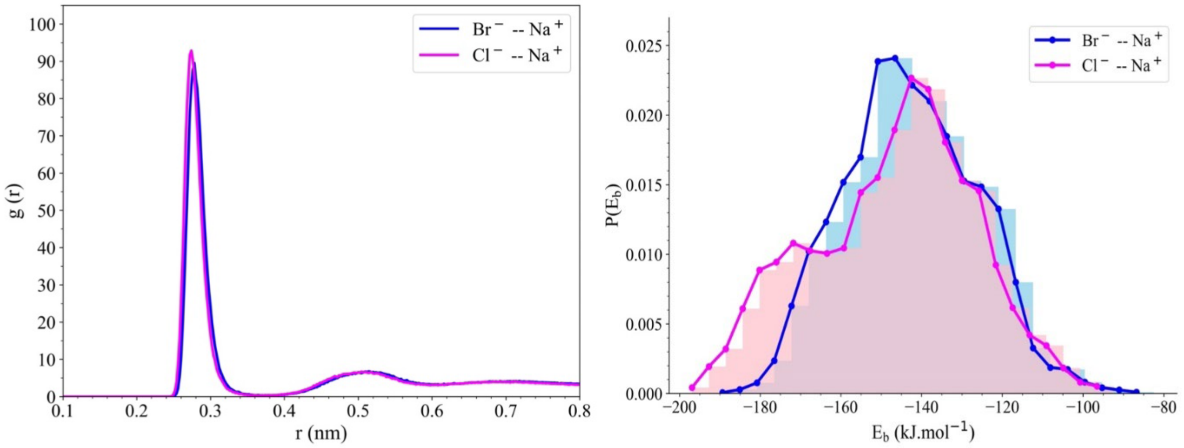

Figure S13: (a) Radial distribution function of Br^- and Cl^- with Na^+ in a pure 1M NaBr/1M NaCl aqueous solution. Energy distribution profile for the NaBr and NaCl interactions. Energies, calculated and normalized based on 50 molecules each of Br^- and Cl^- ions to all Na^+ ions in the aqueous solution, presented in kJ/mol.

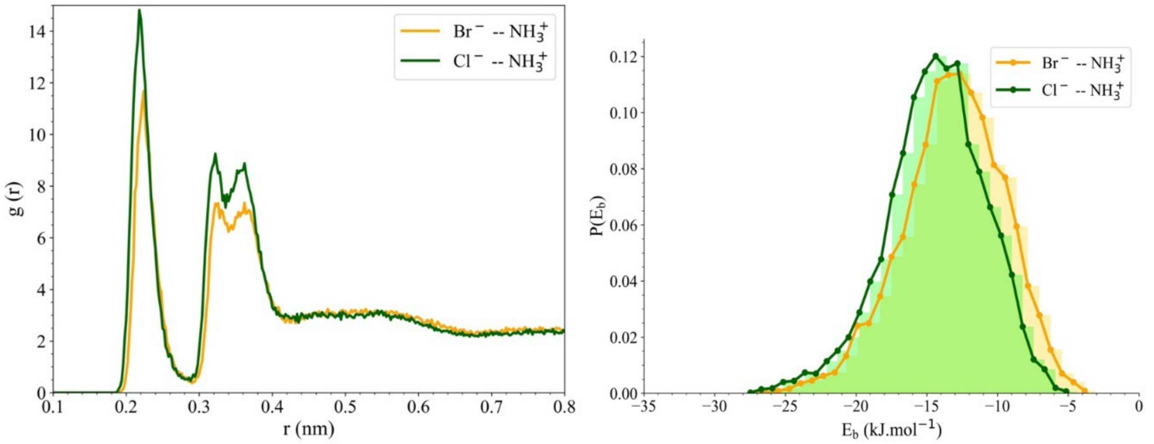

Figure S14: (a) Radial distribution function of Br^- and Cl^- with hexylammonium headgroup (NH_3^+) in mixed 0.5M NaBr/0.5M hexylammonium bromide/1M NaCl aqueous solution. Energy distribution profile for the hexylammonium–Br and hexylammonium–Cl interactions. Energies, calculated and normalized based on 50 molecules each of Br^- and Cl^- ions to all Na^+ ions in the aqueous solution, are presented in kJ/mol.

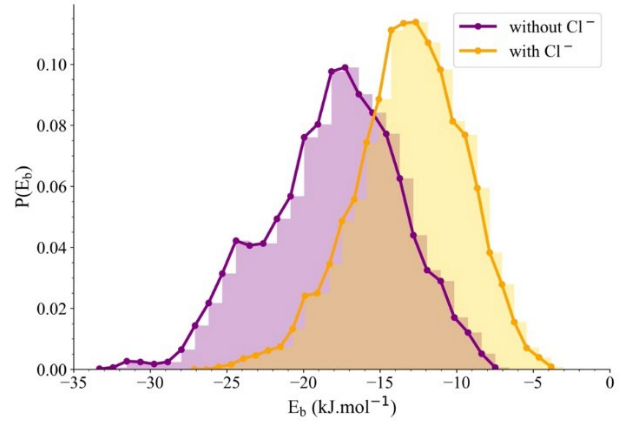

Figure S15: Energy distribution profile for the hexylammonium–Br interactions with and without the inclusion of NaCl. Energies, calculated and normalized based on 50 molecules of Br^- ions to all Na^+ ions in each aqueous solution, are presented in kJ/mol.

References

(1) Brown, M. A.; Redondo, A. B.; Jordan, I.; Duyckaerts, N.; Lee, M.-T.; Ammann, M.; Nolting, F.; Kleibert, A.; Huthwelker, T.; Machler, J.-P.; et al. A new endstation at the swiss light source for ultraviolet photoelectron spectroscopy, x-ray photoelectron spectroscopy, and x-ray absorption spectroscopy measurements of liquid solutions. *Review of Scientific Instruments* **2013**, *84* (7), 073904-073908.

(2) Ekholm, V.; Caleman, C.; Bjärnhall Prytz, N.; Walz, M.-M.; Werner, J.; Öhrwall, G.; Rubensson, J.-E.; Björneholm, O. Strong enrichment of atmospherically relevant organic ions at the aqueous interface: The role of ion pairing and cooperative effects. *Physical Chemistry Chemical Physics* **2018**, *20* (42), 27185-27191, 10.1039/C8CP04525A. DOI: 10.1039/C8CP04525A.

(3) Werner, J.; Persson, I.; Björneholm, O.; Kawecki, D.; Saak, C.-M.; Walz, M.-M.; Ekholm, V.; Unger, I.; Valtl, C.; Caleman, C.; et al. Shifted equilibria of organic acids and bases in the aqueous surface region. *Physical Chemistry Chemical Physics* **2018**, *20* (36), 23281-23293, 10.1039/C8CP01898G. DOI: 10.1039/C8CP01898G.

(4) Perrin, D. D. *Dissociation constants of organic bases in aqueous solution*; Butterworths, 1965.

(5) Bergström, S.; Olofsson, G. Thermodynamic quantities for the solution and protonation of four c6-amines in water over a wide temperature range. *Journal of Solution Chemistry* **1975**, *4* (7), 535-554. DOI: 10.1007/BF00643377.

(6) Lee, M.-T.; Orlando, F.; Khabiri, M.; Roeselová, M.; Brown, M. A.; Ammann, M. The opposing effect of butanol and butyric acid on the abundance of bromide and iodide at the aqueous solution–air interface. *Physical Chemistry Chemical Physics* **2019**, *21* (16), 8418-8427, 10.1039/C8CP07448H. DOI: 10.1039/C8CP07448H.

(7) Chen, S.; Artiglia, L.; Orlando, F.; Edebeli, J.; Kong, X.; Yang, H.; Boucly, A.; Corral Arroyo, P.; Prisle, N.; Ammann, M. Impact of tetrabutylammonium on the oxidation of bromide by ozone. *ACS Earth and Space Chemistry* **2021**, *5* (11), 3008-3021. DOI: 10.1021/acsearthspacechem.1c00233.

(8) Olivieri, G.; Parry, K. M.; D'Auria, R.; Tobias, D. J.; Brown, M. A. Specific anion effects on na+ adsorption at the aqueous solution–air interface: Md simulations, sessa calculations, and photoelectron spectroscopy experiments. *The Journal of Physical Chemistry B* **2018**, *122* (2), 910-918. DOI: 10.1021/acs.jpcb.7b06981.

(9) Shah, A.-u.-H. A.; Ali, K.; Bilal, S. Surface tension, surface excess concentration, enthalpy and entropy of surface formation of aqueous salt solutions. *Colloids and Surfaces A: Physicochemical and Engineering Aspects* **2013**, *417*, 183-190. DOI: https://doi.org/10.1016/j.colsurfa.2012.10.054.

(10) Winter, B.; Faubel, M. Photoemission from liquid aqueous solutions. *Chemical Reviews* **2006**, *106* (4), 1176-1211. DOI: 10.1021/cr040381p.

(11) Shinotsuka, H.; Da, B.; Tanuma, S.; Yoshikawa, H.; Powell, C. J.; Penn, D. R. Calculations of electron inelastic mean free paths. Xi. Data for liquid water for energies from 50 ev to 30 kev. *Surface and Interface Analysis* **2017**, *49* (4), 238-252. DOI: https://doi.org/10.1002/sia.6123.

(12) Habka, S.; Very, T.; Donon, J.; Vaquero-Vara, V.; Tardivel, B.; Charnay-Pouget, F.; Mons, M.; Aitken, D. J.; Brenner, V.; Gloaguen, E. Identification of ion pairs in solution by ir spectroscopy: Crucial contributions of gas phase data and simulations. *Physical Chemistry Chemical Physics* **2019**, *21* (24), 12798-12805, 10.1039/C9CP00700H. DOI: 10.1039/C9CP00700H.

(13) Loche, P.; Steinbrunner, P.; Friedowitz, S.; Netz, R. R.; Bonthuis, D. J. Transferable ion force fields in water from a simultaneous optimization of ion solvation and ion–ion interaction. *The Journal of Physical Chemistry B* **2021**, *125* (30), 8581-8587. DOI: 10.1021/acs.jpcb.1c05303.

(14) Moučka, F.; Nezbeda, I.; Smith, W. R. Chemical potentials, activity coefficients, and solubility in aqueous nacl solutions: Prediction by polarizable force fields. *Journal of Chemical Theory and Computation* **2015**, *11* (4), 1756-1764. DOI: 10.1021/acs.jctc.5b00018.
